# New fossil insect order Permopsocida elucidates major radiation and evolution of suction feeding in hemimetabolous insects (Hexapoda: Acercaria)

**DOI:** 10.1038/srep23004

**Published:** 2016-03-10

**Authors:** Di-Ying Huang, Günter Bechly, Patricia Nel, Michael S. Engel, Jakub Prokop, Dany Azar, Chen-Yang Cai, Thomas van de Kamp, Arnold H. Staniczek, Romain Garrouste, Lars Krogmann, Tomy dos Santos Rolo, Tilo Baumbach, Rainer Ohlhoff, Alexey S. Shmakov, Thierry Bourgoin, André Nel

**Affiliations:** 1 State Key Laboratory of Palaeobiology and Stratigraphy, Nanjing Institute of Geology and Palaeontology, Chinese Academy of Sciences, Nanjing, People’s Republic of China; 2 Staatliches Museum für Naturkunde Stuttgart, Stuttgart, Germany; 3 Institut de Systématique, Évolution, Biodiversité, Muséum national d’Histoire naturelle, Paris, France; 4 AgroParisTech, Paris, France; 5 Division of Entomology, Natural History Museum, and Department of Ecology & Evolutionary Biology, University of Kansas, Lawrence, Kansas, United States of America; 6 Division of Invertebrate Zoology, American Museum of Natural History, New York, New York, United States of America; 7 Charles University, Faculty of Science, Department of Zoology, Prague, Czech Republic; 8 Lebanese University, Faculty of Sciences II, Department of Biology, Beirut, Lebanon; 9 ANKA/ Institute for Photon Science and Synchrotron Radiation, Karlsruhe Institute of Technology (KIT), Eggenstein-Leopoldshafen, Germany; 10 Laboratory for Applications of Synchrotron Radiation, Karlsruhe Institute of Technology (KIT), Karlsruhe, Germany; 11 Im Königsfeld 22A, Saarbrücken, Germany; 12 Arthropoda Laboratory, Palaeontological Institute, Russian Academy of Sciences, Moscow, Russia

## Abstract

With nearly 100,000 species, the Acercaria (lice, plant lices, thrips, bugs) including number of economically important species is one of the most successful insect lineages. However, its phylogeny and evolution of mouthparts among other issues remain debatable. Here new methods of preparation permitted the comprehensive anatomical description of insect inclusions from mid-Cretaceous Burmese amber in astonishing detail. These “missing links” fossils, attributed to a new order Permopsocida, provide crucial evidence for reconstructing the phylogenetic relationships in the Acercaria, supporting its monophyly, and questioning the position of Psocodea as sister group of holometabolans in the most recent phylogenomic study. Permopsocida resolves as sister group of Thripida + Hemiptera and represents an evolutionary link documenting the transition from chewing to piercing mouthparts in relation to suction feeding. Identification of gut contents as angiosperm pollen documents an ecological role of Permopsocida as early pollen feeders with relatively unspecialized mouthparts. This group existed for 185 million years, but has never been diverse and was superseded by new pollenivorous pollinators during the Cretaceous co-evolution of insects and flowers. The key innovation of suction feeding with piercing mouthparts is identified as main event that triggered the huge post-Carboniferous radiation of hemipterans, and facilitated the spreading of pathogenic vectors.

The extraordinary diversity and success of insects is mainly based on two large radiations in Holometabola and Acercaria^1^. The latter lineage includes Hemiptera (true bugs, cicadas, plant lice, whiteflies, and scale insects) and Thripida (thrips), as well as Psocodea (barklice and true lice). Acercarians play a major role in most terrestrial ecosystems, and include numerous important pest species, because of plant-feeding adaptations and/or frequent function as vectors of animal and plant pathogens. Increasing species diversity from barklice to thrips and bugs corresponds to the evolutionary transition from chewing mouthparts to stylet-like sucking-piercing mouthparts. This major transformation represented one of the last remaining enigmas in the evolutionary history of insects, because the phylogeny of Acercaria was still unresolved^2^,^3^,^4^,^5^. Compression fossils of stemgroups of the acercarian orders are known from the Carboniferous to the Cretaceous^1^,^6^,^7^,^8^,^9^, but are not sufficiently preserved to resolve their morphological evolution.

Here we report and describe the new key taxon *Psocorrhyncha burmitica*, based on recently discovered fossils from mid-Cretaceous Burmite amber (Figs 1 and 2). They are related to less-completely known compression fossils, together representing the new order Permopsocida spanning the Permian-Cretaceous.

The monophyly of Acercaria is currently supported by several morphological autapomorphies^5^,^10^, but has been questioned by recent molecular analysis^2^ in which Psocodea appeared as sister group to Holometabola (Supporting Information S1 Text). We propose a new phylogeny of Acercaria, based on morphological characters; some were obtained after the study of *Psocorrhyncha*. Our phylogenetic analysis confirms the monophyly of Acercaria including Psocodea (Fig. 3, Fig. S12), and thus questions the sister group relationship of the latter taxon with Holometabola that was recently proposed in the extensive phylogenomic analysis by the 1Kite project^2^.

We applied an innovative preparation technique (Supporting Information Fig. S1,S1 Text) to the amber fossils, which permitted the examination of the composition of the mouth cone, gut contents, feces, and even sperm of these specimens. Our Scanning Electron Microscopy (SEM) analysis of extracted pollen from the gut contents allowed a determination of angiosperms of the extant family Nyssaceae (tupelo trees) as host plants (Fig. 1).

With the new fossil evidence, we clarify the evolution of feeding modes within this important group of insects. The ‘coned-mouth’ of the Permopsocida is derived from chewing mouthparts of barklice and represented an intermediate step towards the stylet-like mouthparts of thrips and bugs. It also had autapomorphic structures that represented the second original attempt towards realization of a suction feeding mode that lasted for 185 million years. The convergently evolved rostrum of palaeodictyopterids was the first evolutionary experiment for such a feeding mode in insects during the late Paleozoic and existed 320–250 million years ago^6^.

## Results

### Systematic Paleontology

Order Permopsocida Tillyard, 1926 sensu et stat. nov.

#### Included families

Permian to Liassic (with some doubt) Psocidiidae Tillyard, 1926, Permian Permopsocidae Tillyard, 1926, and Jurassic to earliest Upper Cretaceous (with a problematic Permian taxon) Archipsyllidae Handlirsch, 1906, incl. the new archipsyllid genus *Psocorrhyncha*.

#### Emended diagnosis

**Figure 1:**
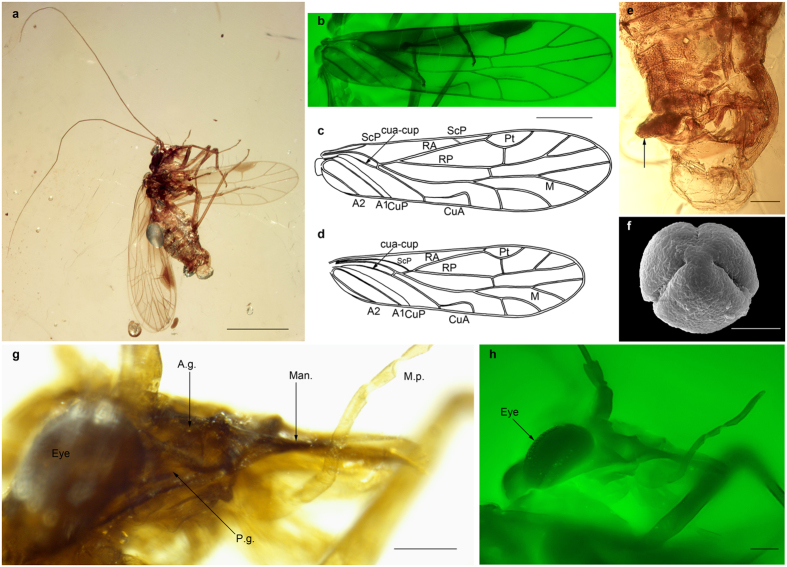
*Psocorrhyncha burmitica* gen. et sp. nov. (Archipsyllidae) from mid Cretaceous Burmese amber, latest record of the new order Permopsocida. Male holotype NIGP161473. (**a**) General habitus. (**b**) Forewing, photomicrograph under green fluorescence. (**c**) Reconstruction of forewing. (**d**) Reconstruction of hind wing (both drawn by PN). (**e**) Apex of abdomen full of pollen grains and fecal pellet (arrow). (**f**) Pollen grain extracted from the abdomen. (**g**) Head, right profile. (H) Head, right profile, photomicrograph under green fluorescence. A1 first anal vein; A2 second anal vein; CuA cubitus anterior; CuP cubitus posterior; M median; Man. mandible; M.p. maxillary palp; A.g. anterior part of gena; P.g. posterior part of gena; RA radius anterior; RP radius posterior; ScP subcosta posterior. Scale bars 1 mm (**a**), 0.5 mm (**b**–**d**), 100 μm (**e**,**g**,**h**), 50 μm (**f**).

**Figure 2:**
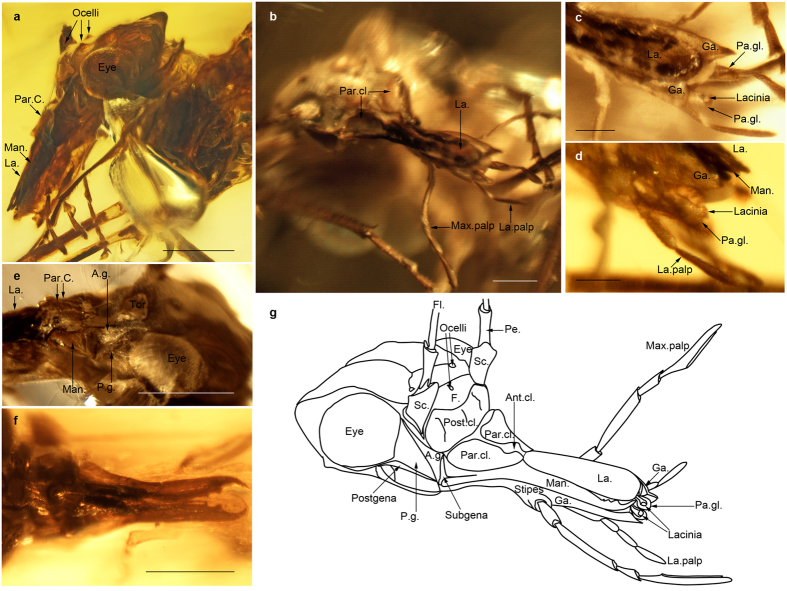
**Head of *Psocorrhyncha burmitica* gen. et sp. nov.** (**a**) Left lateral view. (**b**) Dorso-frontal view. (**c**) Dorsal view, apex of mouthparts. (**d**) Lateral view, apex of mouthparts. (**e**) Lateral view, gena and base of mandible. (**f**) Dorsal view of mandibles. (**g**) Reconstruction of head (drawn by PN). Allotype specimen SMNS Bu-157 (**a**–**e**, **g**); Paratype specimen SMNS Bu-135 (**f**). Ant.cl. median part of anteclypeus; A.g. anterior part of gena; P.g. posterior part of gena; Ga. galea; F. frons; Fl. flagellomere; La. labrum; La. palp labial palp; Man. mandible; Max. palp maxillary palp; Pa.gl. paraglossa; Par.cl. paraclypeus; Pe. pedicel; Post.cl. postclypeus; Sc. scape, Tor. Antennal torulus. Scale bars, 200 μm (**a**,**e**,**f**), 100 μm (**b**), 50 μm (**c**,**d**).

(Figs 1 and 2, Figs S2–6). Head somewhat flattened and depressed; clypeus not strongly swollen; mandibles elongate, with a strong molar plate and a long incisor; four maxillary palpomeres; three labial palpomeres; paraglossae long and sclerotized, appearing as half tubes; paraclypeal lobes present; median part of anteclypeus membraneous; gena divided into two parts by a furrow; ocell-ocular distance < inter-ocellular distance; tarsi four-segmented; fore- and hind wings of similar size, shape, and venation; subcosta posterior ScP present; radius posterior RP two-branched; median vein M normally four-branched (five-branched in one genus); areola postica present; two anal veins present; pterostigmata between costa C and radius anterior RA, of identical shape in all wings; RA forming a pronounced posterior curve below pterostigmata; radius R with a pronounced angle at level of base of M; M + CuA basally fused with R, separating from radius far from wing base; long crossvein cua-cup present between cubitus posterior CuP and cubitus anterior CuA; abdomen with strong basal constriction; cerci absent; female ovipositor well-developed and sclerotized.

Family Archipsyllidae Handlirsch, 1906.

### *Psocorrhyncha burmitica* gen. et sp. nov

#### Type species of genus

*Psocorrhyncha burmitica* sp. nov.

#### Material

Male holotype NIGP161473 and male paratype NIGP161474 at Nanjing Institute of Geology and Paleontology (NGIP, Academia Sinica, China); female allotype SMNS Bu-157 and female paratype SMNS Bu-135 at State Museum for Natural History in Stuttgart (SMNS, Germany).

#### Type locality

Hukawng Valley, Kachin State, Myanmar (Burma). The exact outcrop among the various amber mines in this valley is unknown, because the specimens were acquired from traders.

#### Type horizon

Burmese amber (Burmite)^11^,^12^, Earliest Upper Cretaceous, earliest Cenomanian, absolute age 98.79 ± 0.62 million years ago (mya) established by U-Pb dating of zircons from the rind of the unprocessed amber^13^. Nuclear magnetic resonance spectra and the presence of araucaroid wood fibers in amber samples indicate an araucarian (possibly *Agathis*) tree as source for the resin^14^.

#### Etymology

The generic name refers to the resemblance of this taxon with the Psocodea and its affinities with the Hemiptera (old name Rhynchota). The gender of the name is feminine. The specific epithet refers to the country of origin.

#### Diagnosis

Forewing ScP short, ending on C at level of base of M + CuA and re-emerging distally as a faint phantom-vein ending on R (the fusion of forewing ScP with C is a character present in the other Archipsyllidae as putative synapomorphy, but it is re-emerging as a distinct vein in these genera, instead of being phantom-like); hind wing ScP fused with R.

#### Comment

*Psocorrhyncha burmitica* is the youngest fossil record of Archipsyllidae. A redescription of the enigmatic Permian psocidiid species *Dichentomum tinctum* Tillyard, 1926, and a discussion of all other taxa previously attributed to Permopsocida is provided online in the Supporting Information (S1 Text).

### Description

The description is based mainly on holotype NIGP161473, completed by information from the three other fossils.

Body 2.4 mm long between apex of abdomen and base of antennae, and glabrous; head with rostrum 0.9 mm long; head capsule 0.4 mm long; occiput abruptly bent; compound eyes well developed, 0.28 mm wide and well separated; dorsal part of head between compound eyes divided in two parts by weak furrow: a posterior part (looking like a corypha of Fulgoromorpha^15^,^16^), divided into two pronounced lobes each bearing a smooth but pronounced lobe, separated by a median sulcus; and a vertical anterior part (looking like a metopa of Fulgoromorpha^15^) anterior of compound eyes, bearing two well-separated lateral ocelli, each being closer to eye than to other ocellus; anterior ocellus positioned far from lateral ocelli, on a line separating dorsal part of head from frons (Fig. 2g, Fig. S3e); frons narrow, as long as narrow sclerotized postclypeus, which is separated from anteclypeus by a furrow; anteclypeus short, 0.4 times shorter than labrum, composed by two lateral parts (paraclypea), rounded elongate, more sclerotized and higher than membranous median part (Fig. 2b,g, Fig. S3b); mouthparts hypognathous but clearly movable relative to head capsule (as documented by forming different angles with head capsule in different specimens) (Fig. 1a, Fig. S3a, Fig. S4a,b); labrum elongate, 0.28 mm long, three times as long as wide, apically spatulate and rounded, flat and thin, with small apical setae; mandibles elongate, 0.29 mm long and 0.09 mm wide at base (paratype specimen NIGP161474), three times as long as wide at base, with a broad base and distal two-thirds narrow; molar plates well developed bearing three distinct teeth on left mandible and only two on right mandible; incisor far from molar plate, with a strong apical tooth and two smaller basal teeth (Fig. 2f, Fig. S2a,h); anterior condyle of mandible connected with latero-basal angle of paraclypeus (Fig. S2a,e); posterior condyle connected to distal margin of gena; gena large and broadly quadrangular with transverse furrow dividing it obliquely, anterior part distinctly concave, bearing condyle of mandible; posterior part more convex than anterior part (Fig. 2e,g), apparently bearing a small sensilla along its posterior margin below compound eye (paratype specimen NIGP161474); subgena between anterior part of gena and mandible; postgena between gena and maxilla (Fig. S2b); maxillary palps long with four palpomeres (Fig. 1g, Fig. 2a,b,g, Fig. S4b), apical palpomere long, 0.18 mm long, subapical palpomere 0.07 mm long, shorter than apical palpomere and with an apical bevel cut, basal palpomere short, 0.18 mm long, second palpomere as long as apical one, 0.17 mm long; cardo and stipes well separated, articulation of maxilla visible^17^; lacinia long, as long as galea, spoon-like, i.e., broadened in its distal part but apically narrowed and without subapical tooth, detached from stipes and deeply inserted into head (Fig. 2c,d,g, Fig. S2c,d); galea broader than lacinia, with distal half broadened, apex bearing short setae, distally ending close to apex of mandible, apically serving as guide for mandibles due to ‘T-profile’ cross-section (Fig. S2c,d,g,); three labial palpomeres (Fig. 2c,d,g), with basal palpomere shortest, 0.05 mm long, second palpomere 0.1 mm long, third palpomere 0.09 mm long; labium with elongate prementum and half-tube-shaped paraglossae as guide for laciniae; antennae inserted well below compound eyes, well separated, with a subquadrate scape 0.11 mm long and 0.10 mm wide, pedicel as long as scape but narrower (Fig. S2g, Fig. S3e, Fig. S4b); 14 elongate flagellomeres, finely annulated, with individual lengths decreasing progressively toward apex; first, second, and third flagellomeres bearing an apical, elliptical flat sensilla (Fig. S5a,b), and first flagellomere bearing also a basal one; membraneous zone between flagellomeres simple, without mechanism for rupturing antennae (as in Psocodea^18^); no sclerotized ring at base of first flagellomere in cavity of pedicel; scape inserted on head capsule by a dicondylic articulation (acute lateral antennifer and weaker, median articulation point on head capsule, see Fig. S2g); no cephalic trichobothria.

Prothorax developed as narrow neck bearing an anterior sclerotized ring with small indentations and posterior part desclerotized (Fig. 2a); mesothorax and metathorax higher than prothorax, separated by subvertical pleural furrow; mesothoracic scutum deeply concave; wings inserted high on meso- and metathorax; tegula present at forewing base.

Legs long and thin; profemur 0.5 mm long, protibia 0.7 mm long, protarsus 0.4 mm long; mesofemur 0.5 mm long, mesotibia 0.7 mm long, mesotarsus 0.4 mm long; metafemur not enlarged, 1.3 mm long, 0.1 mm wide, metatibia 0.9 mm long, 0.03 mm wide, metatarsus 0.6 mm long; tibiae with two strong apical spurs and a row of spines; 4-segmented tarsi (Fig. S4e,g); tarsomeres bearing a row of spines, tarsomeres without plantulae; strong apical pretarsal claws without basal tooth, a fleshy and broad arolium present between pretarsal claws (Fig. S4f).

Forewing and hind wing elongate, of nearly same size and shape; forewing 2.6 mm long, 0.7 mm wide; ScP ending on costal margin C 0.5 mm from wing base, and re-emerging 0.3 mm distally to reach radius R as a phantom-vein (Fig. S6c); area between R and C broad, 0.17 mm wide; R, M, and CuA fused into a common stem at wing base, making a weak posterior curve for 0.52 mm; then M + CuA and R separating, with R and basal stem R + M + CuA forming a pronounced angle at this point (Fig. 1b,c); RP and RA separating 0.15 mm distal of base of M + CuA; convex RA with pronounced posterior curve surrounding darkly pigmented pterostigma, 0.42 mm long and 0.14 mm wide, pterostigma basally delimited by a vein (Fig. S6b); a crossvein perpendicular to RA and to RP exactly below middle of pterostigma; concave RP with only one distal fork, 1.3 mm from its base; M and CuA separating immediately distal of point of re-emergence of M + CuA, or CuA emerging directly on stem R + M + CuA just basal of base of M (depending on specimen); neutral stem of M long, 0.85 mm long before first fork; anterior branch of M with a deep fork distally and branches ending near wing apex (but in paratype specimen NIGP161474, this vein is simple in one wing while it is forked in the second); posterior branch of M with a more open fork and shorter branches ending on posterior wing margin; convex CuA short before crossvein cua-cup terminates on it, cua-cup aligned with distal part of CuA; distal part of CuA long, 0.5 mm long before areola postica; areola postica long and narrow, parallel to posterior wing margin, with CuA1 curved and CuA2 short; cua-cup weaker than CuA and M, 0.40 mm long between base of CuP and CuA (Fig. 1b,c); concave CuP weakly curved and simple; two convex simple anal veins basally curved. Forewing articulation partly visible in specimen NIGP161473: humeral plate (HP) and basisubcostale (BSc) united but well separated from basiradiale (BR) and second axillary sclerite (2Ax) by two deep furrows that extend transversely from wing base and tegula (Fig. S6a).

Hind wing 2.3 mm long, 0.71 mm wide; nearly identical to forewing, with following differences: wing narrower, with narrower pterostigma; ScP longer than in forewing, ending on R 0.52 mm from wing base (Fig. 1d); area between R and costal margin C much narrower than in forewing, 0.11 mm wide; cua-cup weak, ending on M + CuA; stem of M + CuA relatively long distal of its separation from radius, 0.14 mm long; areola postica very faint with CuA1 phantom-like.

A strong constriction between thorax and abdomen present due to small first abdominal segment, bearing small lateral lobes (Fig. 1a, Fig. S3a,c); sternum I not visible. Abdomen ca. 1.3 times as long as thorax plus head; abdominal terga short and of nearly same length; cerci absent.

Male appendages symmetrical (Fig. S5c), with a large, sclerotized spoon-like hypandrium; a short epiproct partly hidden by a fecal pellet (composed of pollen) extended from anus, and two, long subvertical paraprocts, 0.23 mm long, with a subbasal hook, a trichobothrial field on external surface of epiproct; aedeagus large, 0.25 mm long, broadly triangular, with three small, lateral spines; endosoma extruded exhibiting ductus ejaculatorius and gonopore II; hypandrium (sternite IX) long, spoon-like, 0.37 mm long; some sperm is visible in the abdomen.

Female ovipositor curved upwards (Fig. S3d,f), with ventral valvulae (gonapophyses VIII) with ventral margin bearing small denticles and a dorso-apical part bearing a raking structure; dorsal valvulae (gonapophyses IX) triangular, narrow, and elongate, ending with a small upward denticle, and less sclerotized than ventral valvulae; gonoplacs broad and weakly sclerotized, with an apical lobe; gonocoxites VIII large, broadly quadrangular in an anterior position; gonocoxite IX triangular and small at base of gonoplacs; epiproct and paraprocts of same length, shorter than gonoplacs, pointed at apices; tergum X longer than tergum IX; laterotergite VIII with a distal membraneous zone; subgenital plate with two broad arms; sternum IX reduced; tergum IX + X narrow; trichobothrial field on a gibbosity of epiproct.

### Phylogenetic analysis

**Figure 3:**
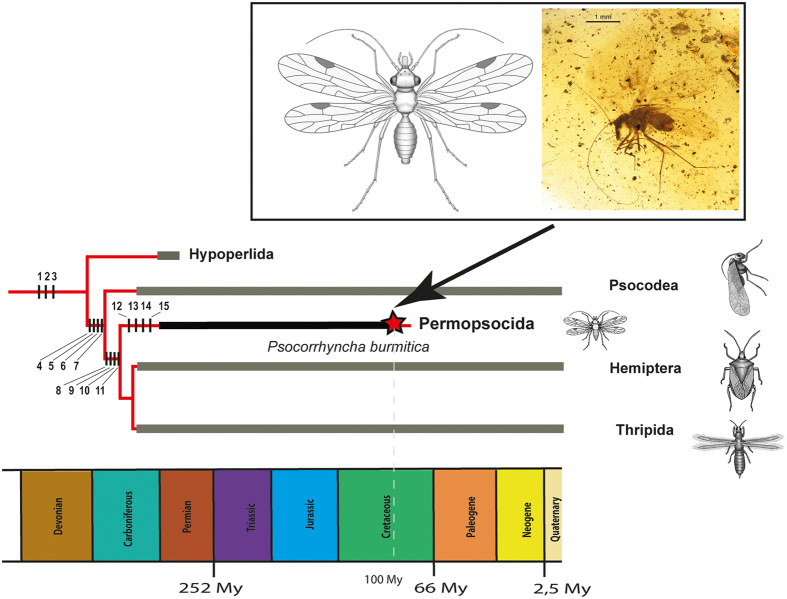
Phylogeny of Acercaria (drawn by RG). List of synapomorphic characters. Clade Acercaria: characters ‘1’ (common stem R + M + CuA), ‘2’ (neutral crossvein cua-cup between concave CuP and convex CuA), ‘3’ (elongate lacinia). Clade [Psocodea + (Permopsocida + (Thripida + Hemiptera))]: characters ‘4’ (clypeus divided by a furrow into ante- and postclypeus, but a character variable in Pterygota), ‘5’ (maxillary lacinia not in direct contact with stipes), ‘6’ (cerci absent), ‘7’ (reduction of number of tarsomeres to four or less). Clade [Permopsocida + (Thripida + Hemiptera)]: characters ‘8’ (paraclypeal lobes present), ‘9’ (labrum elongate), ‘10’ (mentum elongate and sclerotized), ‘11’ (gena divided into two lobes). Clade Permopsocida: characters ‘12’ (ocell-ocular distance < inter-ocellular distance), ‘13’ (tarsi four-segmented), ‘14’ (pterostigma in hind wing limited by costal wing margin and a deep posterior curve of vein RA), and ‘15’ (abdominal segment 1 narrow and reduced).

We conducted a cladistic analysis using morphological data to correctly place crucial fossil taxa and resolve the relationships within Acercaria (Hypoperlidae, Psocodea, Permopsocida, Thripida, and Hemiptera). Therefore, mainly those morphological characters that are also discernible in the fossils have been selected. The data matrix used for the analysis consists of 16 taxa (four outgroup taxa in Polyneoptera and Holometabola, and 12 of the ingroup, see Table S2) and 62 characters (see Table S3). The characters were treated as non-additive and unordered. The matrix was constructed with WinClada ver. 1.00.08 (see Table S4) and analysed with the parsimony software package TNT^19^. Using New Technology search method with default parameters resulted in a single topology, presented in Fig. S12, and the resulting acercarian phylogeny in Fig. 3. Its length is 100 steps, CI = 0.730, and RI = 0.833. The Bremer support of subclades are indicated in Fig. S12. This tree is slightly better resolved than the strict consensus tree of the two most parsimonious trees resulting from Traditional search method with default options. It supports a monophyletic Acercaria with Hypoperlidae as sister group of all other Acercaria; Permopsocida resolves as sister group of Thripida + Hemiptera (Condylognatha), and Psocodea as sister group of Permopsocida + Condylognatha. The new fossil genus and species *Psocorrhyncha burmitica* is recovered within the monophyletic Permopsocida as sister group of *Archipsylla*.

The results of our phylogenetic analysis agree with most other recent studies^3^,^5^ in the relationships among the extant acercarian orders. However, there is one important difference to the most recent, extensive phylogenomic analysis of insects by the 1Kite project^2^, which proposed a paraphyletic Acercaria with Psocodea as sister group of Holometabola. The authors of the 1Kite project remarked, ‘convincing morphological features and fossil intermediates supporting a monophyly of Acercaria are lacking’. Contrarily to the op cite analysis, Acercaria monoplyly is well recovered and supported by a large set of morphological autapomorphies, even if some of these characters are unknown in some fossil groups like Permopsocida or absent in early stem group representatives like Hypoperlidae^1^,^10^. These characters include the following: postclypeus large and with large cibarial dilator muscles; asymmetrical mandibles; laciniae transformed into stylet-like, slender rods, detached not directly connected to stipes and retractile, withdrawn deep into head capsule (a complex and strong character!); labial palps reduced (max. three palpomeres) or lost; cibarial pump (with similar sclerites and muscles especially in Psocodea and Thysanoptera); presence of an areola postica at least in forewings (character subject to reversions); neutral crossvein cua-cup between concave CuP and convex CuA, weaker than CuA; a common stem R + M + CuA at wing base; 1st abdominal sternum strongly reduced or absent; cerci completely reduced (one-segmented in Hypoperlidae); abdominal ganglia concentrated in a single ganglionic mass; max. four malpighian tubules; biflagellate spermatozoa; and acrosome of spermatozoa without perforatorium (last three characters not observable in fossils). We therefore assume that the 1Kite result concerning the phylogenetic position of Psocodea could be due to a systematic error (e.g. long branch attraction) or methodological artefact.

Remark. The reduction of the number of tarsomeres to max. four is no longer an acercarian apomorphy as there are five in Hypoperlidae.

## Discussion

**Figure 4:**
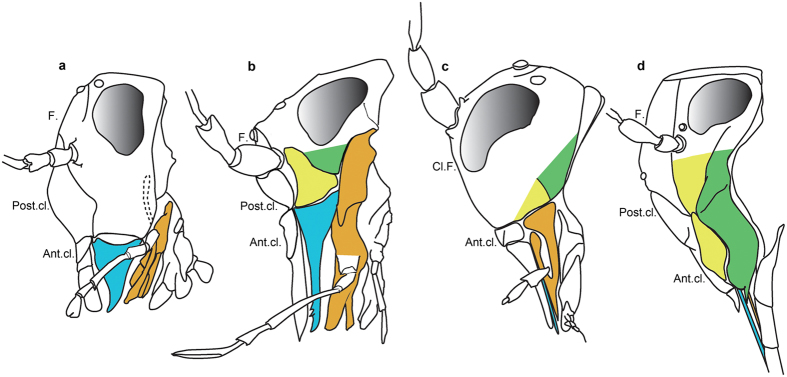
Hypothesis of head and mouthpart morphologies in Acercaria (drawn by TB and PN). (**a**) Psocodean groundpattern (also present in Hypoperlidae). (**b**) Permopsocidan groundpattern. (**c**) Thripidan groundpattern, reconstructed after the head of an adult Tubulifera, and *Moundthrips*. (**d**) Hemipteran groundpattern. Mandible: blue; maxilla: brown; anterior part of gena (mandibular lobe): yellow; posterior part of gena (maxillary lobe?): green. Ant.cl. anteclypeus; Cl.F. clypeo-frons; F. frons; Post.cl. postclypeus.

The gena of *Psocorrhyncha* gen. nov. and other Permopsocida is subdivided by a strong furrow into a dorsal and ventral lobe, unlike in Psocodea, Permian Hypoperlidae (Supporting Information), and non-acercarian insects (Figs 1g,h, 2e,g and 4). The dorsal lobe is posteriorly adjacent to the antennal insertion, and the ventral lobe is not fused with the maxilla. Adults of the Mesozoic thripidan genus *Moundthrips* (Fig. S13b), extant thripidan young nymphs, and adults of the thripidan suborder Tubulifera have the same lobes^20^,^21^,^22^,^23^, but they are no longer visible in adult Terebrantia. We consider the dorsal lobes as possibly homologous to the hemipteran mandibular plates (lora), supporting their parietal origin^24^,^25^,^26^. The hemipteran maxillary plate is in the same position as the ventral lobe of the gena in *Psocorrhyncha* and Thripida, suggesting a possible composite origin in part of genal (parietal) origin and in part of stipital (appendicular) origin. Both hypotheses for the origin of the maxillary plate are currently proposed^24^,^25^,^26^,^27^,^28^. These subdivisions of the gena were developed in Permopsocida possibly to strengthen this crucial sclerite as a support for a mandible stronger than in Hypoperlidae and Psocodea. To further strengthen the feeding mechanism, the permopsocid head also has an elongate prementum and half-tube-shaped paraglossae serving as guiding device for the laciniae. In Hemiptera mandibular and maxillary plates developed similarly, closing laterally the mouth cone base, while the mandibular plate plus the maxilla provide the same function in Thripida. A rudimentary mouth ‘cone’ is already present in Permopsocida, even if laterally opened. This intermediary condition provides a possible scenario of the transformation from chewing to sucking-piercing mouthparts in Acercaria. The permopsocid head (Fig. 4) can be interpreted as a less efficient precursor of the highly derived labial cone of the Thripida + Hemiptera (Fig. 2c,d,g), with its transformation of mandibles and laciniae into very thin stylets, deeply inserted into the head capsule, as well as the strongly modified gutter-like labium in Hemiptera. These last changes opened the possibility for adaptation to a wide range of different food sources: on pollen, but also on plant or animal tissues or fluids.

The sclerotized paraclypeal lobes and membranous medial part of the anteclypeus of Permopsocida (Fig. 2b,g, Fig. S3b) and Thripida suggest that the ability for rotation of mouthparts to guide the mouthparts to food^29^ is a ground plan condition for Condylognatha. In Hemiptera, the paraclypeal lobes are maintained, while the anteclypeus is no longer membranous but secondarily sclerotized to serve as muscle attachment for the cibarial pump^30^.

**Figure 5:**
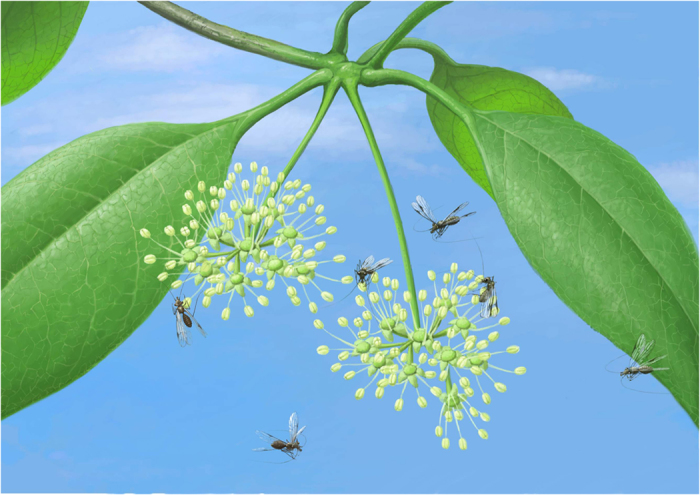
Life history reconstruction of *Psocorrhyncha burmitica* gen. et sp. nov., from the Late Albian epoch of Burmese amber. Specimens depicted as flying or feeding on flowers of Nyssaceae (drawn by DH).

Hypoperlidae and Permopsocida were feeding on pollen organs of seed ferns and gymnosperms during the Permian, but at least the youngest Cretaceous representative, *Psocorrhyncha*, adapted to the floral changes occurring between the Permian and the Cretaceous and fed on angiosperm pollen grains (Fig. 5, Supporting Information Fig. S1 and S1 Text). Hypoperlidae, Psocodea, and Permopsocida can swallow entire palynomorphs^31^,^32^, but the elongation of the mouthparts into a rudimentary ‘cone’ (elongation of the labrum, mandibles, and maxilla, paraglossae serving as guiding device for the laciniae, galea apically serving as guides for mandibles) in Permopsocida possibly also allowed for suction feeding on nectar thanks to their long laciniae, and chewing plant tissue thanks to their acute mandibles with strong molar plates. The mouthparts of Thripida and Hemiptera became more modified through development of a closed mouth cone and elongate stylets to pierce cells^22^,^32^, tissues, and vessels of plants and animals. This allowed for the exploitation of numerous new food resources, which at least partly explains their significant diversification since the Permian^2^. The development of highly modified piercing mouthparts facilitated the evolution of an increasing number of pathogenic vectors in Hemiptera (and to a lesser extent Thripida), because they are able to introduce viruses and bacteria deeper into plant or animal tissues and vessels than Acercaria with chewing mouthparts (i.e. Psocodea) can do.

Hypoperlidae and Permopsocida must be at least of the same Late Carboniferous age as Psocodea and Thripida + Hemiptera^10^,^33^,^34^ (Fig. 3), even though their oldest known fossils are recorded from the Early Permian^2^,^6^. Acercaria still had a low diversity in the Carboniferous, with less than ten known species^34^. The Hypoperlidae apparently were never very diverse, with only four Permian genera with about 13 species, while the Permopsocida are divided into three families with 25 known species ranging from the Lower Permian to the beginning of the Upper Cretaceous. Unlike Hypoperlidae, psocodeans could survive and diversify during the Middle Jurassic-Cretaceous^35^, probably because of their alimentation as omnivorous scavengers on plant and animal remains, algae, and lichens. However, Psocodea never reached the high level of diversity characteristic for Hemiptera. These latter insect order already greatly diversified early in the Permian, Triassic, and the Jurassic^2^,^33^. Today it includes about 82.000 living species. A comparative analysis of species numbers in relation to feeding modes, phylogenetic position, and stratigraphic range suggests that mouthpart specialization for suction feeding was the key innovation that explains the huge post-Carboniferous radiation within Acercaria (Table S5).

Permopsocids could survive during the Triassic and Jurassic but had to face competition from numerous other pollenivorous insects, such as thrips, flies, and long-tongued scorpionflies^36^. The final extinction of Permopsocida during the mid-Cretaceous, after having existed for at least 185 million years, was most probably influenced by the Cretaceous diversification of angiosperm flowers, correlated with obligatory insect pollination^36^. This promoted the evolution of numerous new groups of competing pollenivorous pollinators within beetles, moths, flies, and bees^2^,^37^.

Thus, the paleontological evidence suggests an explanation for the huge radiation within Acercaria and the extinction of less diverse stem clades in relation to mouthpart specialization and plant-insect co-evolution.

## Materials and Methods

The amber specimens were ground and polished manually and with polishing machines. The holotype was embedded in Canada balsam to make the inclusion more clearly visible. Pollen was extracted from the gut content of the holotype with a Pasteur pipette, washed with toluene, and then photographed using SEM. Fossil specimens were studied with different stereo microscopes, light microscopes, and laser confocal microscopes, partly with green fluorescence as light source. Microphotographs were made with digital cameras, and focus stacking software was used to increase depth of field. All images were processed with Adobe Photoshop^TM^. Synchrotron micro-computer tomography (X-ray micro-CT) scans were performed at the TOPO-TOMO beamline of the ANKA Synchrotron Radiation Facility of the Karlsruhe Institute of Technology. A more detailed account on materials and methods is available online in the Supporting Information (S1 Text).

## Additional Information

**Data Availability**: The ZooBank LSID (Life Science Identifier) for the new genus and species is as follows: Psocorrhyncha burmitica LSID, urn:lsid:zoobank.org:pub:A38DB5C5-BCBA-4906-8723-F5CFAA067F34.

**How to cite this article**: Huang, D.-Y. *et al.* New fossil insect order Permopsocida elucidates major radiation and evolution of suction feeding in hemimetabolous insects (Hexapoda: Acercaria). *Sci. Rep.* **6**, 23004; doi: 10.1038/srep23004 (2016).

## Supplementary Material

Supplementary Information
