# Supplementary material for: New fossil insect order Permopsocida elucidates major radiation and evolution of suction feeding in hemimetabolous insects (Hexapoda: Acercaria)
Source: Sci Rep. 2016 Mar 10;6:23004. doi: 10.1038/srep23004 (PMC4785345; doi:10.1038/srep23004)
Supplement: Supplementary Information [file srep23004-s1.pdf]

SUPPLEMENTARY INFORMATION

Title

New fossil insect order Permopsocida elucidates major radiation and evolution of suction feeding in hemimetabolous insects (Hexapoda: Acercaria)

Short Title

New fossil order elucidates evolution of Acercaria

Authors

Di-Ying Huang[1]‡, Günter Bechly[2]‡, Patricia Nel[3,4]‡, Michael S. Engel[5,6], Jakub Prokop[7], Dany Azar[8], Chen-Yang Cai[1], Thomas van de Kamp[9,10], Arnold H. Staniczek[2], Romain Garrouste[3], Lars Krogmann[2], Tomy dos Santos Rolo[9], Tilo Baumbach[9,10], Rainer Ohlhoff[11], Alexey S. Shmakov[12], Thierry Bourgoin[3], and André Nel[3]*

S1 Text

(A) Extended Material and Methods. (B) Systematic Paleontology. (C) SI References.

(A) Extended Material and Methods

Specimen depositories

MCZ - Museum of Comparative Zoology at Harvard University, Cambridge, USA

NHM - The Natural History Museum London, UK

NIGP - Nanjing Institute of Geology and Paleontology, Academia Sinica, China

PIN - Paleontological Institute of Russian Academy of Sciences, Moscow, Russia

PU - Perm State University, Perm, Russia (specimen stored at PIN)

SMNS - Staatliches Museum für Naturkunde Stuttgart (SMNS), Germany

Preparations of specimens

The holotype of *Psocorrhyncha burmitica* gen. et sp. nov. (NIGP161473) is embedded in a large piece of amber containing several syninclusions (more than 20 arthropods). The amber piece was cut to separate each inclusion. The piece containing the holotype was subsequently ground to remove excess amber and then polished. Following this procedure, we found the included insect specimen was not clearly visible resulting from a series of fractures in the amber, causing mirror effects. In addition, there was a large bubble enveloping the abdomen (including genitalia), and a large portion of the thorax and wings.

To remedy these optical disturbances we infused the amber piece with Canada balsam. First, the specimen was manually polished using Emery papers with varying and successively finer grains until the apices of the fractures were reached (Fig. S1a). The polished piece was then immersed into Canada balsam and slowly heated until boiling (Fig. S1b), a procedure repeated several times until all fractures were infilled with the Canada balsam, rendering a clear view of the specimen. To clear the obscured view created by the bubble, the specimen was polished again to minimize the distance between bubble and amber surface (Fig. S1c). The amber was punctured manually with a thin (size '00') entomological pin, which had been previously modified so that its apex was flattened and sharpened like a chisel (acting as a miniaturized drill bit) (Fig. S1d). Afterwards, the specimen was immerged again into Canada balsam and heated gently until the resin filled the bubble. Once completed, the preparation was left for two days to permit the Canada balsam to enter the inclusion, clear it, and set. The final result perfectly revealed all internal structures of the insect as well as the pollen grains that fill much of the abdomen.

Specimens SMNS Bu-135 and SMNS Bu-157 of *P. burmitica* were prepared using Struers Dap-6 and LaboPol-4 grinding and polishing machines. These specimens were not coated nor embedded in artificial resin to avoid disturbances during µCT scanning.

Extraction of pollen grains from abdomen of holotype NIGP161473

After the Canada balsam settled uniformly inside the insect's body, the result was an appearance similar to that of an extant insect treated with potassium hydroxide (KOH), allowing a detailed observation of internal structures and gut contents (pollen grains of *Nyssapollenites*). To extract some of these pollen grains, the cuticle was pierced with a minuten pin, with a hook-like tip, mounted to a handle. The pin was used to pierce the abdomen of the insect and then turned smoothly to scrape the internal surface and dislodge some of the pollen grains (Fig. S1e). The narrowed tip of a drawn-out Pasteur pipette was then introduced into the abdomen adjacent to detached pollen grains. Repeated pumping allowed extraction of some palynomorphs (Fig. S1f). Subsequently, the pollen grains were washed with toluene to eliminate all residues of Canada balsam and then isolated with a pin and mounted for SEM study with a Tescan Vega LSU scanning electron microscope at the MNHN.

Examination of fossils with 3D X-ray micro-computer tomography

Searching for preserved internal morphological characters inside the amber inclusions, we applied 3D X-ray micro-computer tomography with synchrotron radiation (micro-CT)[1-3]. Scans were performed at the TOPO-TOMO beamline[4] of the ANKA Synchrotron Radiation Facility at Karlsruhe Institute of Technology (KIT). The parallel-beam tomographic scans covered an angular range of 180°, measured using a filtered polychromatic beam with a spectral peak at about 15 keV. Under such experimental conditions conventional absorption

contrast and phase contrast (in the so-called edge-enhancement regime) are the physical image formation mechanisms. An indirect detector system composed of a 12µm LSO:Tb scintillator, diffraction limited optical microscope (Optique Peter) and 12 bit pco.dimax high speed camera (2016 x 2016 pixels resolution) was employed to capture 3000 projections per tomographic scan with an exposure time of 10 ms each. A 5x optical magnification led to an effective pixel size of 2.44 µm.

Prior to volume reconstruction, all projection images were processed with the phase retrieval ImageJ plugin ANKAphase[5]. Volume reconstruction was done by the PyHST software developed by the European Synchrotron Radiation Facility, Grenoble, France, and KIT[6].

Specimen SMNS Bu-157, even though appearing perfectly preserved under light microscopy, did not give any image contrast with µCT under any parameters (e.g. phase contrast).

Specimen SMNS Bu-135 gave contrast, but even here the remaining internal structure had a relative poor quality only allowing an incomplete reconstruction of the mandibles and maxillae. The results indicate that internal morphological characters were not (SMNS Bu-157) or only partly (SMNS Bu-135) preserved, with the interesting exception of pollen inside the gut of the latter specimen (Fig. S1g-h). One possible explanation for the poor results may be that specimen Bu-157 was fully enfused with resin prior to fossilization, as observed in various other insect inclusions before. In this case, intensity modulations would occur only on the surface of the specimen. However, since all modalities of X-ray CT are volumetric, contrast in the tomographic reconstruction can only be observed if the change in the complex refractive index occurs in a volume comparably as large as a voxel. For visible light observations, interference based reflections are visible even from surface structures, as evidenced by the interference from a few nm thin oil film on water. We suspect that an

analogous mechanism is responsible for the lack of contrast for the X-ray tomography in the present case.

Observation of fossils with microscopy

SMNS Bu-135 and SMNS Bu-157 were studied at SMNS with a Leica M80 stereo-microscope and 1.6* Plan Achromat lens. Photographs were taken with a Leica DFC490 digital macro camera on a Leica Z16-Apo Macroscope.

All specimens from NIGP (Nanjing, China) and SMNS (Stuttgart, Germany) were loaned and examined at the MNHN (Paris, France) using Olympus SZX-9 and Nikon SMS-1500 stereomicroscopes. Photographs were taken with a Canon D550 digital camera with reverse lens MP-E 65mm, and line drawings prepared using a camera lucida. Original photographs were processed using Adobe Photoshop™ CS4.

Observations and photographs of the specimens at NIGP were taken using a Zeiss Discovery V20 stereomicroscope and a Zeiss Axio Imager 2 light microscope with an attached digital camera. Some photomicrographs were taken using green fluorescence as a light source attached to a Zeiss Axio Imager-2 light microscope and confocal laser scanning microscopy (CLSM) Zeiss LSM 710 with ×10 objectives and 488 nm laser.

The compression fossils from MCZ and NHM were examined with Nikon SMZ 645 and Wild M5 stereomicroscopes in a dry state and under a thin layer of ethanol. Photographs were taken using a Canon D550 digital camera with MP-E 65mm lens and processed with Adobe Photoshop™ CS4.

Most microphotographs were generated from focus stacks using the Helicon Focus Pro software, apart from the SMNS specimens for which Leica Application Suite 3.8.0 was used for focus stacking.

(B) Systematic Paleontology

Revision of Permopsocida Tillyard, 1926

Standard wing venation terminology was employed throughout the descriptions as it has been applied to representatives of Acercaria[7]. We elevate the previous psocodean suborder Permopsocida to ordinal rank, revise the permopsocidan families, and redescribe the crucial psocidiid species *Dichentomum tinctum* Tillyard, 1926.

Clade Acercaria Börner, 1904

Definition. Acercaria Börner, 1904 comprises Psocodea (including 'Psocoptera' and Phthiraptera), Thripida (including Thysanoptera), and Hemiptera. The order Zoraptera has been considered as sister group of Acercaria and both taxa have been classified together as Paraneoptera[8,9]. However, polyneopteran affinities of Zoraptera recently gained further support[10-12], so that Paraneoptera either has to be rejected as polyphyletic[11] or considered as synonymous with Acercaria[13]. We herein add the extinct order Permopsocida and the family Hypoperlidae to Acercaria.

Order Permopsocida Tillyard, 1926 stat. nov. (= Permopsocina Tillyard, 1926)[14]

Stratigraphic range. Permopsocida are relatively frequent in Permian outcrops[15], but the clade is also known from Liassic, Middle Jurassic, and Lower Cretaceous outcrops. *Psocorrhyncha* gen. nov. from the earliest Upper Cretaceous is the latest occurrence of and only known amber representative of Permopsocida.

Included families. Permian to Liassic (with some doubt) Psocidiidae Tillyard, 1926, Permian Permopsocidae Tillyard, 1926, and Jurassic to earliest Upper Cretaceous (with a problematic Permian taxon) Archipsyllidae Handlirsch, 1906, incl. the new archipsyllid genus

Psocorrhyncha. Cyphoneuridae Carpenter, 1932 (with *Cyphoneura* Carpenter, 1932; *Australocypha* Tillyard, 1935; *Lophiocypha* Tillyard, 1935) were later included in Permopsocida[16], but more recently demonstrated to belong to Thripida[17]. Likewise, the family Edgariekiidae Jell and Duncan, 1986 (*Edgariekia una* Jell and Duncan, 1986), originally placed in Permopsocida[18], is a junior synonym of the thripidan family Lophioneuridae Tillyard, 1921[17].

Family Archipsyllidae Handlirsch, 1906

Stratigraphic and geographic range. Permian?, Jurassic to earliest Upper Cretaceous.

Emended diagnosis. The venation of the previously described Archipsyllidae agrees with that of *Psocorrhyncha*, with the following two exceptions: subcosta posterior ScP basally reaching the costal margin and distally re-emerging to end into radius anterior RA basal of pterostigma, not only in the forewings, but also in the hind wings; longer areola postica reaching the level of the pterostigma. This special shape of the ScP in forewings is a putative synapomorphy of the Archipsyllidae, even if this character is convergently present in a few modern Psocodea of the family Lepidopsocidae. The Archipsyllidae with bodies (partly) preserved (*A. sinica*, *E. sojanense*) share with *Psocorrhyncha* elongate mouthparts, with long and narrow labra, long laciniae with one apical tooth, male genitalia with a large hypandrium, four-segmented tarsi, simple and symmetrical pretarsal claws, large arolia, and flagellomeres annulate and long.

Included genera. *Archipsylla* Handlirsch, 1906, *Archipsyllodes* Vishniakova, 1976, *Archipsyllopsis* Vishniakova, 1976, *Eopsylla* Vishniakova, 1976, and *Psocorrhyncha* gen. nov.

Family Psocidiidae Tillyard, 1926 sensu nov.

Stratigraphic and geographic range. Permian; Australia, Russia and USA.

Composition. This family previously comprised five genera, only two of which can be accurately considered as Permopsocida, viz. *Dichentomum* Tillyard, 1926 and *Stenopsocidium* Tillyard, 1935.

Emended diagnosis. Fore- and hind wing with similar venation; ScP long, ending on RA distal of base of radius posterior RP in all wings; RP two-branched; media vein M four-branched; areola postica longer than high; no crossvein between M and first branch of cubitus anterior CuA1. At least *Dichentomum* has small crossveins between costa C and ScP.

Dichentomum tinctum Tillyard, 1926

Redescription. The genus *Dichentomum* and its type species *D. tinctum* rank among the better preserved and complete of the Permian Permopsocida, but have not been re-examined since the original description by Tillyard[14] and the two revisions by Carpenter[19-20]. A comparison with the amber material of *Psocorrhyncha* offered a unique opportunity to detect and verify crucial characters for *Dichentomum*. This complementary study is based on specimens 3324a, 3331a-b, 3348, 3323a-b, and 3347a-b (all at MCZ). The following important characters supplement the previous descriptions: head in lateral view more flat than in *Psocorrhyncha* and without a strong angle between posterior and anterior parts of dorsal side; frons narrow, as long as a narrow sclerotized postclypeus, which is separated from anteclypeus by a furrow; compound eyes well developed and well separated; two well-separated lateral ocelli, each closer to compound eye than to other ocellus; anterior ocellus hardly visible but situated far from lateral ocelli; antennae inserted well below compound eyes, well separated from each other, with a subquadrate scape, pedicel as long as scape but narrower; exact number of flagellomeres undeterminate, but all of them long and finely annulated; *Dichentomum* has certainly not 50 short antennomeres, contra Carpenter[20]

(flagellomeres are finely annulated and Carpenter obviously misinterpreted the annulations as flagellomeres); anteclypeus short, distinctly shorter than labrum, with two lateral parts (paraclypeus), rounded elongate (Fig. S8a); labrum elongate, ca. two times as long as wide, apically rounded and flat; mandibles elongate, ca. three times as long as wide at base, with a broad base and distal two-thirds narrow; molar plate possibly visible, but incisor teeth not visible; anterior condyle of mandible visible, connected with latero-basal angle of paraclypeus; gena large and broadly quadrangular with a transverse furrow dividing it obliquely into anterior (mandibular plate) and posterior (maxillary plate) parts (Fig. S8c), subgena between anterior part of gena and mandible; three labial palpomeres, with basal palpomere shortest, second palpomere longest, third palpomere slightly shorter than second palpomere; maxillary palps long, four palpomeres (Fig. S8a), apical palpomere long, subapical palpomere shorter than apical palpomere, basal palpomere relatively short, second palpomere as long as apical palpomere; lacinia and galea long, overlapping apices of mandibles, apically narrowed and without visible subapical tooth (Fig. S8d); reconstruction of wing venation proposed by Carpenter[20] accurate, in particular in presence of a series of short crossveins between C and ScP, at least in forewing (Nel et al.[7] re-analysed the pattern of wing venation of *Dichentomum* and considered it to be of acercarian type); legs long and thin; tibiae with two apical spurs (Fig. S8c,d); all tarsi four-segmented; tarsomeres without plantulae; strong pretarsal claws without subapical tooth (Fig. S8b); arolium between pretarsal claws not visible; a strong constriction between thorax and abdomen due to small first abdominal segment (Fig. S8c); cerci absent (confirmation of Carpenter[20]); ovipositor well developed with ventral valvulae (gonapophyses VIII) with ventral margin bearing at least small denticles.

Family Permopsocidae Tillyard, 1926

Stratigraphic and geographic range. Permian, USA.

Emended diagnosis. Fore- and hind wing with similar venation; ScP long, ending on RA distal of base of RP in all wings; RP two-branched; M four-branched; areola postica higher than long; a crossvein between M and CuA1.

Remark. The family Permopsocidae currently comprises four genera (see Table S1), i.e. *Permopsocus* Tillyard, 1926, *Lithopsocidium* Carpenter, 1932, *Orthopsocus* Carpenter, 1932, and *Progonopsocus* Tillyard, 1926.

Redefinition of Hypoperlidae Martynov, 1928

As indicated by Shcherbakov[21], the Permopsocida (*Dichentomum*) have a forewing venation similar to those of some taxa (especially *Boreopsocus* Shcherbakov, 1994) currently attributed to the Permian family Hypoperlidae. Thus it is crucial to discuss the composition and phylogenetic relationships of Hypoperlidae.

Rasnitsyn[22] included seven genera in the Permian family Hypoperlidae: *Hypoperla* Martynov, 1928, *Hypoperlopsis* Zalessky, *Martynopsocus* Karny, 1930, *Kaltanelmoa* Rohdendorf, 1961, *Fatjanoptera* Martynova, 1961, *Tshunicola* Rasnitsyn, 1977, and *Tshekardobia* Rasnitsyn, 1977. Shcherbakov[21] restricted the Palaeozoic Hypoperlidae to embrace the four genera *Hypoperla*, *Idelopsocus* Zalessky, 1929, *Kaltanelmoa*, and *Boreopsocus* Shcherbakov, 1994.

The venation of *Hypoperla elegans* Martynov, 1928 (type species of Hypoperlidae, type family of the order Hypoperlida) is typical for Acercaria by having a common stem R+M+CuA, M+CuA separating from R distally; convex CuA immediately emerging from M+CuA; long crossvein cua-cup between concave cubitus posterior CuP and CuA, concave near CuP and convex near CuA, CuA, with an areola postica (see Figs. S9c-d). The only other group having a common stem R+M+CuA is Archaeorthoptera. But, Archaeorthoptera have

CuA with a higher number of distal branches and a concave anterior branch of CuP ending on convex CuA instead of a cua-cup[30]. Nevertheless, *H. elegans* differs from Permopsocida in several important plesiomorphies: RP with a series of parallel posterior branches instead of a single fork, as in modern Acercaria and Permopsocida (a likely plesiomorphy because numerous posterior branches of RP are known in the ground plans of polyneopterous orders and in Neuropterida and Panorpida); no distinct angle of radius at base of M+CuA; pterostigma more 'rudimentary' and consisting of a darker zone covering apical parts of ScP, RA, and apical part of area between RA and RP, not delimited posteriorly by RA. The same pattern occurs in *Hypoperla grata* Novokshonova, 1998 and *Hypoperla vaulevi* Novokshonov, 2001.

The venation of *Idelopsocus tataricus* Zalessky, 1929 is clearly acercarian, showing a convex CuA emerging with concave M from a common stem with R, a long brace cua-cup between concave CuP and CuA, concave near CuP and convex near CuA, and two convex simple anal veins. The CuA of *I. tataricus* is simple, concave ScP ends on RA, and concave RP and M both have three branches with few crossveins. This venation is closer to modern Acercaria than to that of *Hypoperla*. It differs from *Psocorrhyncha* in lacking a strong angle between RA and basal stem R+M+CuA, and not having a sclerotized pterostigma. *Idelopsocus diradiatus* Rasnitsyn, 1996 also has a venation closer to non-hypoperlid Acercaria in that the RP only has two branches, and M with only three branches, but lacking any angle in the course of R at base of M+CuA. *Idelopsocus diradiatus* has a forked CuA, unlike *I. tataricus*. *Idelopsocus tataricus* and *I. incommendatus* Novokshonov et al., 2002 share similar venation characters except for presence of an areola postica. The venation is somewhat variable among the *Idelopsocus* species, especially the number of main vein branches. Unlike *Hypoperla*, where only the distal parts of the wings have darkened membranes, species of *Idelopsocus* possess sclerotized pterostigmata in fore- and hind wings

(Figs. S9f and S11a-b)[15], not homologous to that of Permopsocida because the pterostigmata cover a zone crossing the distal area between the anterior wing margin and RA and part of the area between RA and RP. In Permopsocida, the pterostigmata are delimited posteriorly by RA. *Idelopsocus mutovinus* Rasnitsyn and Aristov, 2013 is probably also a Hypoperlidae, although the basal part of the vein CuA is not clearly visible. *Idelopsocus diradiatus* and *Idelopsocus splendens* (Zalessky, 1948) have five-segmented tarsi (specimens PIN 1700/3298 or PU 2/129 attributed to *I. splendens* by Novoskshonov[24] and Rasnitsyn[15]), while the type specimen of *I. splendens* is an isolated wing originally described as *Hypoperlopsis splendens*. This tarsal character is a plesiomorphic in Acercaria and most insects.

Boreopsocus has a venation most suggestive to that of Permopsocida, with RP having a distal fork, pterostigmata in fore- and hind wings delimited by a posterior curve of RA, with a crossvein below it and RP (but narrower than in Permopsocida, except *Stenopsocidium*). Unlike Permopsocida[21], it lacks an angular R, and possesses five-segmented tarsi. *Kaltanelmoa sibirica* (based on the basal two-thirds of an isolated wing) also has a venation typical of Acercaria (courses of M and cubital veins, simple fork of CuA). RP and M in this species appear to be simply forked, as in modern acercarians and Permopsocida, but R lacks an angle in its course distal to base of M. The area of the putative pterostigma is hardly preserved.

In summary, the Hypoperlidae *sensu* Shcherbakov[21] appear to be a 'group' of acercarian genera, but lack a clear apomorphy that could support them as a clade. They may represent a paraphyletic 'evolutionary grade' (with regard to wing venation and number of tarsomeres) from *Hypoperla* to *Boreoposocus* sharing several apomorphies with Permopsocida (similar pterostigmata and venation). The venation of *Idelopsocus* could represent an 'intermediate' stage, having reduced branchings in RP and M, compared to the situation observed in *Hypoperla*, but with a particular pterostigma different from *Boreopsocus*

and Permopsocida. Interestingly, a strikingly similar phenomenom happened during the evolution of the odonatopteran pterostigmata: the basal clades (Meganisoptera) have no pterostigma, whereas Odonata have a pterostigma delimited posteriorly by RA. The pterostigma in the 'intermediate' clade Protanisoptera is almost identical in shape and position to that of *Idelopsocus*[25].

The wing venation of Hypoperlidae lacks any synapomorphy with the palaeodictyopteran groups (Dictyoneuridea sensu Rasnitsyn[15]). In particular the common stem R+M+CuA, present in the Hypoperlidae and the Acercaria, is absent in palaeodictyopteran orders. Also, Hypoperlidae has only two convex simple anal veins, identical to Acercaria, but different from the anal veins of Palaeodictyoptera, where there are numerous anal veins reinforced by a prominent anal ridge (the so-called 'anal brace'). This neopteran family cannot be considered as a member of a grade that would have given rise to these palaeopterous insects.

Rasnitsyn[15] considered the mouthparts as diagnostic characters for the order Hypoperlida. He described them as 'chewing though often beak-like elongate, with lacinia rod- or styletlike, clypeus convex indicating strong cibarial muscles, or, if flat, mandibles and laciniae long, jointly forming short beak'. Such structures are barely visible in the few described Hypoperlidae with preserved bodies. In fact, the mouthparts of *Idelopsocus splendens* (specimens PIN 1700/3298 and PU 2/129), *Idelopsocus diradiatus*, and *Idelopsocus galinae* Novokshonov, 2001 are not particularly elongate and resemble the mouthparts of Psocodea, especially in the non-divided gena (see Fig. S11d).

Rasnitsyn[15] considered the piercing rostrum of Palaeodictyoptera and Hemiptera as homologous and derived from a hypoperlidan ancestor. Kukalová-Peck[26] presented a detailed reconstruction of palaeodictyopteroid mouthparts, with structures (lacinia, ante- and postclypeus, mandibular condyles, etc.) generally unavailable for observation in fossils, or

undissected modern insects. Other interpretations by Kukalová-Peck[27], Laurentiaux[28], or even Dohrn[29], remain more reasonable, describing very long stylet-like mandibles, and long maxillary palps, but without information on other parts such as laciniae. Even though these structures are reminiscent of those of Hemiptera (except presence of maxillary palps), they are certainly the result of convergence as already proposed by Laurentiaux[16] and Emeljanov[30], and are not synapomorphies with those Acercaria with piercing mouthparts. All other structures (especially the wing venation) exhibit no synapomorphies between Palaeodictyoptera and Acercaria.

Redescription of the hypoperlid *Idelopsocus splendens* (Zalessky, 1948)

A re-examination of two specimens PIN 1700/3298 and PU 2/129 attributed to *I. splendens* by Novoskshonov[24] and Rasnitsyn[15] revealed the following characters: head without a clear subdivision into sub-horizontal posterior part and subvertical anterior part bearing ocelli; flagellomeres numerous, relatively short, apparently annulated; ocelli present (two visible) on vertex; compound eyes large; clypeus apparently not subdivided into ante- and postclypeus; paraclypeal lobes absent; mouthparts short; labrum not elongate; mandibles strong and psocodean-like; maxillary palps long, five palpomeres; lacinia elongate, not guided by paraglossa nor by galea at its apex, as in Psocodea, but exact structure cannot be recognized; division between cardo and stipes probable, but not clearly visible; labium short with short prementum and paraglossae not half-tube-shaped; labial palps not clearly visible; gena not divided into two lobes (Fig. S11d); tarsi five-segmented, no tarsal plantulae (Fig. S11c); pretarsal claws strong with arolium between them; wings homonomous; venation of acercarian-type with a common stem R+M+CuA and a crossvein cua-cup between CuP and CuA; M re-emerging from R well distal of wing base, forked twice into four branches, M1-M2 and M3-M4; RP forked; radial stem lacking pronounced posterior angle; two anal veins;

pterostigmata present on all wings, but not posteriorly delimited by R; areola postica present, longer than wide; shape of ScP unclear in all wings; presence or absence of abdominal sternum I cannot be verified; first abdominal segment narrower than others, but less than in *Psocorrhyncha*; female abdominal terga IX, X and XI completely developed; cerci present, short and unsegmented (Figs. S11e-f); ovipositor present and well developed; male genital structures unknown.

Alimentation of Permopsocida and Hypoperlidae

The guts of three specimens of *P. burmitica* (specimens SMNS Bu-157, NIGP161473, and SMNS Bu-135) are filled with one morphotype of pollen grains, which are mostly intact and untampered. A fecal pellet extruding from the abdomen of specimen NIGP161473 is also totally composed of the same type of pollen grains (Fig. S5c).

Some grains were extracted from the abdomen of NIGP161473 and examined with SEM (see Material and Methods). The morphology of these grains corresponds with fossil *Nyssapollenites* and extant members of the angiosperm family Nyssaceae[31]. A unique difference is their smaller size (diameter: 11-14 µm for fossil vs ca. 30 µm for extant species of *Davidia*, 40 µm for species of *Camptotheca*, and 46 µm for species of *Nyssa*[32]). Presence of intact, unopened pollen grains in the guts and feces of these specimens of *Psocorrhyncha* suggests the pollen wall might have been infiltrated with digestive enzymes, as in extant bees[33].

Thus, imagos of *Psocorrhyncha* fed on entire pollen grains, without masticating them with their well-developed molar plates. Moreover, it appears their elongate mouthparts were not adapted for chewing nyssacean flowers with their short and flat corollae. As these insects belong to hemimetabolous Acercaria, their nymphs certainly had similar mouthpart morphology and diets as the adults.

Extant Nyssaceae only bloom during a brief period in spring (April to June). Mouthparts of Permopsocida are completely different from those of typical modern, exclusive pollen-feeding insects that visit flowers having short corollae (*e.g.,* beetles of the lineages Scarabaeidae, Leiodidae, or Staphylinoidea, in which the mandibles have reduced incisors, but with brush-like hairs on their lacinia and galea[34-35], and most certainly different from those bees that visit short-corolla flowers). Perhaps adults and nymphs of *Psocorrhyncha* fed upon another food source (*e.g.*, ripened fruits of Nyssacea, or even small insects) during other periods of the year, or the flowering phenology of fossiliferous Nyssaceae differed from that of their extant representatives. In comparison, the mirid predator *Macrolophus pygmaeus* uses pollen as alternative or supplementary food source, favouring nymphal development[36]. Also some insectivorous modern Chrysopidae can be found with the gut full of pollen[37].

One fossil specimen of *Archipsylla sinica* Huang et al., 2008 also has structures tentatively interpretable as sporangia in its gut (Fig. S7). Among the Permian Permopsocida, *Dichentomum tinctum* and *Stenopsocidium elongatum* have elongate mouthparts, similar to those of *Psocorrhyncha* and *A. sinica*. This would suggest that all of these had similar modes of alimentation. However, if Permian permopsocids fed on pallinomorphs, these must have certainly been of a different type than those eaten by *Psocorrhyncha,* as angiosperms did not exist during the Permian. Krassilov et al.[38] found pallinomorphs in the gut of the Middle Permian psocidiid *Dichentomum* (*Parapsocidium*) *uralicum* (Zalessky, 1937). The Permian psocidiid *Dichentomum* (*Parapsocidium*) *uralicum* appeared to have been polylectic[38] (pollen grains of seed ferns and of gymnosperms in its gut), while *Psocorrhyncha* was apparently oligolectic on angiosperm Nyssaceae.

Some modern Psocodea, Thripida, and Hemiptera also feed (in part) on pollen grains. While thrips and hemipterans empty the grains of pollen[39], booklice ingest whole or crushed grains. Gut contents of extant Psocodea can contain angiosperm and gymnosperm pollen

grains, frequently mixed with fungal spores[40]. Interestingly, Krassilov et al.[41] stated, “In the Kungurian of Tchekarda we found taeniate pollen grains in the gut compressions of *Idelopsocus* (Hypoperlidae), … , while *Idelopsocus diradiatus* Rasnitsyn fed on both *Lunatisporites* and *Protohaploxypinus*”. These types of pallinomorphs of these plants are currently assigned to Pterydophyta, plants present in the Lower Permian.

As Hypoperlidae belong to the stem group of Acercaria, and Permopsocida to the stem group of Condylognatha (Thripida+Hemiptera), palynivory seems to be a ground plan character of Acercaria, which evolved dramatically after the Late Palaeozoic.

(C) SI References

1. Perreau, M., Tafforeau, P. Virtual dissection using phase-contrast X-ray synchrotron microtomography: reducing the gap between fossils and extant species. Syst. Entomol. **36**: 573−580 (2011). doi: 10.1111/j.1365-3113.2011.00573.x.
2. Riedel, A. *et al*. a new subfamily of fossil weevils (Coleoptera, Curculionoidea, Attelabidae) and the use of synchrotron microtomography to examine inclusions in amber. Zool. J. Linn. Soc. **165**: 773−794 2012). doi: 10.1111/j.1096-3642.2012.00825.x.
3. van de Kamp, T., dos Santos Rolo, T., Baumbach, T., Krogmann, L. Scanning the past - synchrotron X-ray microtomography of fossil wasps in amber. Entomol. Heute **26**: 151−160 (2014).
4. Rack, A. *et al*. The micro-imaging station of the TopoTomo beamline at the ANKA synchrotron light source. Nucl. Instr. Meth. Phys. Res. (B) **267**: 1978–1988 (2009). doi: 10.1016/j.nimb.2009.04.002.

5. Weitkamp, T., Haas, D., Wegrzynek, D., Rack, A. ANKAphase: software for single-distance phase retrieval from inline X-ray phase-contrast radiographs. J. Synchrotron Radiat. **18**: 617–629 (2011). doi: 10.1107/S0909049511002895.
6. Chilingaryan, S., Kopmann, A., Mirone, A., dos Santos Rolo, T. A GPU-based architecture for real-time data assessment at synchrotron experiments. IEEE Trans. Nucl. Sci. **58**: 1447−1455 (2011). doi: 10.1109/TNS.2011.2141686.
7. Nel, A. *et al.* Traits and evolution of wing venation pattern in paraneopteran insects. J. Morphol. **273**: 480–506 (2012). doi: 10.1002/jmor.11036.
8. Hennig, W. Insect phylogeny. Chichester: Wiley and Sons (1981).
9. Beutel, R.G., Weide, D. Cephalic anatomy of *Zorotypus hubbardi* (Hexapoda: Zoraptera): new evidence for a relationship with Acercaria. Zoomorphol. **124**: 121–136 (2005). doi: 10.1007/s00435-005-0117-z.
10. Letsch, H.O., Simon, S. Insect phylogenomics: new insights on the relationships of lower neopteran orders (Polyneoptera). Syst. Entomol. **38**: 783–793 (2013). doi: 10.1111/syen.12028.
11. Friedemann, K., Spangenberg, R., Yoshizawa, K., Beutel, R. G. Evolution of attachment structures in the highly diverse Acercaria (Hexapoda). Cladistics **30**: 170–201 (2013). doi: 10.1111/cla.12030.
12. Wipfler, B., Pass, G. Antennal heart morphology supports relationship of Zoraptera with polyneopteran insects. Syst. Entomol. **39**: 800–805 (2014). doi: 10.1111/syen.12088.
13. Li, H. *et al*. Higher-level phylogeny of paraneopteran insects inferred from mitochondrial genome sequences. Sci. Rep. **5**: 8527 (2015). doi: 10.1038/srep08527.
14. Tillyard, R. J. Kansas Permian insects. 8. Copeognatha. Am. J. Sci. **11**: 314–349 (1926).

15. Rasnitsyn, A. P. Cohors Cimiciformes Laicharting, 1781. In: Rasnitsyn, A. P, Quicke, D. L. J. editors. History of Insects. Dordrecht: Kluwer Academic Publishers : pp. 104−157 (2002).

16. Laurentiaux, D. Classe des insectes (Insecta Linné, 1758). In: Piveteau, J. editor. Traité de Paléontologie, vol. 3. Paris: Masson & Cie: pp. 397–527 (1953).

17. Nel, P. *et al*. From Carboniferous to Recent: wing venation enlightens evolution of thysanopteran lineage. J. Syst. Palaeontol. **10**: 385–399 (2012). doi: 10.1080/14772019.2011.598578.

18. Jell, P.A., Duncan, P. M. Invertebrates, mainly insects, from the freshwater, Lower Cretaceous, Koonwarra fossil bed, (Korumburra Group), South Gippsland, Victoria. Mem. Assoc. Austral. Palaeontol. **3**: 111–205 (1986).

19. Carpenter, F. M. The Lower Permian insects of Kansas. Part 5. Psocoptera and addition to the Homoptera. Am. J. Sci. **24**: 1–22 (1932).

20. Carpenter, F. M. The Lower Permian insects of Kansas. Part 6. Delopteridae, Protelytroptera, Plectoptera and a new collection of Protodonata, Odonata, Megasecoptera, Homoptera and Psocoptera. Proc. Am. Acad. Arts Sci. **68**: 411–503 (1933).

21. Shcherbakov, D. Y. A new genus of the Paleozoic order Hypoperlida. Russian Entomol. J. **3**: 33–36 (1994).

22. Rasnitsyn, A. P. New Paleozoic and Mesozoic Insects. Paleontol. J. **11**: 60–72 (1977).

23. Béthoux, O., Nel, A. Venation pattern and revision of Orthoptera sensu nov. and sister groups. Phylogeny of Palaeozoic and Mesozoic Orthoptera sensu nov. Zootaxa **96**: 1–88 (2002).

24. Novokshonov, V. G. New insects (Insecta: Hypoperlida, Mischopterida, Jurinida) from the Lower Permian of the Middle Urals. Paleontol. J. **32**: 46–53 (1998).

25. Huguet, A. *et al*. Preliminary phylogenetic analysis of the Protanisoptera (Insecta: Odonatoptera). Geobios **35**: 537–560 (2002). doi: 10.1016/S0016-6995(02)00071-2.

26. Kukalová-Peck, J. Fossil history and the evolution of hexapod structures. In: Naumann, I. D. editor. The Insects of Australia, a textbook for students and research workers, vol. 1. 2nd ed. Melbourne: Melbourne University Press: pp. 141–79 (1991).

27. Kukalová-Peck, J. Unusual structures in the Paleozoic insect orders Megasecoptera and Palaeodictyoptera with description of a new family. Psyche **79**: 243–268 (1972). doi: 10.1155/1972/98019.

28. Laurentiaux, D. Présence d'un rostre eugereonien chez le paléodictyoptère *Stenodictya lobata* Brongniart. Affinites des protohemipteres. C. R. Acad. Sci. Paris **234**: 1997–1999 (1952).

29. Dohrn, A. *Eugereon boeckingi*, eine neue Insectenform aus dem Todtliegenden. Palaeontographica **13**: 333–340 (1866).

30. Emeljanov, A. F. Evolutionary scenario of rostrum formation in the Rhynchota. Entomol. Rev. **82**: 1197–1206 (2002).

31. Traverse, A. Paleopalynology. 2nd ed. Series Topics in Geobiology, vol. **28**. Dordrecht: Springer (2007).

32. Chao, C. Y. Comparative pollen morphololology of the Cornaceae and allies. Taiwania **5**: 93–106 (1954).

33. Roulston, T. H, Cane, J. H. Pollen nutritional content and digestibility for animals. Plant Syst. Evol. **222**: 187–209 (2000).

34. Betz, O., Thayer, M. K., Newton, A. F. Comparative morphology and evolutionary pathways of the mouthparts in spore-feeding Staphylinoidea (Coleoptera). Acta Zool. **84**: 179–238 (2003). doi: 10.1046/j.1463-6395.2003.00147.x.

35. Karolyi, F., Gorb, S. N., Krenn, H. W. Pollen grains adhere to the moist mouthparts in the flower visiting beetle *Cetonia aurata* (Scarabaeidae, Coleoptera). Arthrop.-Plant Interact. 3: 1–8 (2009). doi: 10.1007/s11829-008-9052-5.

36. Vandekerkhove, B., De Clercq, P. Pollen as an alternative or supplementary food for the mirid predator *Macrolophus pygmaeus*. Biol. Control. **53**: 238–242 (2010). doi: 10.1016/j.biocontrol.2010.01.005.

37. Duelli, P. Honigtau und stumme Gesänge: Habitat- und Partnersuche bei Florfliegen (Neuroptera, Chrysopidae). Stapfia **60**: 35–48 (1999).

38. Krassilov, V. A., Rasnitsyn, A.P., Afonin, S. A. Pollen morphotypes from the intestine of a Permian booklouse. Rev. Palaeobot. Palyno. **106**: 89–96 (1999). doi: 10.1016/S0034-6667(99)00002-0.

39. Dicke, F. F., Jarvis, J. L. The habits and seasonal abundance of *Orius insidiosus* (Say) (Hemiptera-Heteroptera: Anthocoridae) on Corn. J. Kansas Entomol. Soc. **35**: 339–344 (1962).

40. Broadhead, E., Richards, A. M. The Peripsocidae and Psocidae (Psocoptera) of East Africa. Syst. Entomol. **5**: 357–397 (1980). doi: 10.1111/j.1365-3113.1980.tb00421.x.

41. Krassilov, V. A., Rasnitsyn, A. P, Afonin, S. A. Pollen eaters and pollen morphology: co-evolution through the Permian and Mesozoic. Afr. Invertebr. **48**: 3–11 (2007).

42. Handlirsch, A. Die fossilen Insekten und die Phylogenie der rezenten Formen. Ein Handbuch für Paläontologen und Zoologen. Leipzig: Engelmann (1906-1908).

43. Enderlein, G. Zur Kenntnis frühjurassicher Copeognathen und Coniopterygiden und über das Schicksal der Archipsylliden. Zool. Anz. **34**: 770–776 (1909).

44. Ansorge, J. Insekten aus dem Oberen Lias von Grimmen (Vorpommern, Norddeutschland). N. Paläontol. Abh. **2**:1–132 (1996).

45. Vishniakova, V. N. Relict Archipsyllidae (Insecta: Psocoptera) in the Mesozoic fauna. Paleontol. J. **10**: 180–188 (1976).

46. Rasnitsyn, A. P. *et al*. Important new insect fossils from Carrizo Arroyo and the Permo-Carboniferous faunal boundary. Bull. New Mexico Mus. Nat. Hist. Sci. **25**: 215–246 (2004).

47. Carpenter, F. M. Superclass Hexapoda. In: Moore, R.C., Kaesler, R. L. editors. Treatise on Invertebrate Paleontology, (R), Arthropoda 4, 3/4. Boulder, Lawrence: Geological Society of America, University of Kansas: xxii + 655 pp. (1992).

48. Tillyard, R. J. Upper Permian insects of New South Wales. 3. The order Copeognatha. Proc. Linn. Soc. N. S. W. 60: 265–279 (1935).

49. Jell, P. A. The fossil insects of Australia. Mem. Qld. Mus. **50**: 1–124 (2004).

50. Carpenter, F. M. The Lower Permian insects of Kansas. Part 8. Additional Megasecoptera, Protodonata, Odonata, Homoptera Psocoptera, Plecoptera, and Protoperlaria. Proc. Am. Acad. Arts Sci. **73**: 29–70 (1939).

51. Moritz, G. Zur Morphologie und Anatomie des Fransenflüglers *Aeolothrips intermedius* Bagnall. 1. Mitteilung: der Kopf. Zool. Jb. Anat. **107**: 557–608 (1982).

52. Seeger, W. Funktionsmorphologie an Spezialbildungen der Fühlergeissel von Psocoptera und anderen Paraneoptera (Insecta); Psocodea als monophyletische Gruppe. Z. Morphol. Tiere **81**: 137–159 (1975).

53. Heming, B. S. Antennal structure and metamorphosis in *Frankhniella fusca* (Hinds) (Thripidae) and *Haplothrips verbasci* (Osborn) (Phlaeothripidae) (Thysanoptera). Quaest. Entomol. **11**: 25–68 (1975).

54. Matsuda, R. Morphology and evolution of the insect head. Mem. Am. Entomol. Inst. **4**: 1–334 (1965).

55. Gewecke, M. Bewegungsmechanismus und Gelenkrezeptoren der Antennen von *Locusta migratoria* L. (Insecta, Orthoptera). Z. Morphol. Tiere **71**: 128–149 (1972).

56. Staudacher, E. M., Gebhardt, M., Durr, V. Antennal movements and mechanoreception: neurobiology of active tactile sensors. Adv. Insect Physiol. **32**:49–205 (2005). doi: 10.1016/S0065-2806(05)32002-9.

57. Badonnel, A. Recherche sur l'anatomie des psoques. Suppl. Bull. Biol. Fr. Belg. **18**: 1–241 (1934).

58. Nel, P. *et al*. Redefining the Thripida (Insecta: Paraneoptera). J. Syst. Palaeontol. **12**: 865–878 (2014). doi: 10.1080/14772019.2013.841781.

59. Dmitriev, D. A. Homologies of the head of Membracoidea based on nymphal morphology with notes on other groups of Auchenorrhyncha (Hemiptera). Eur. J. Entomol. **107**: 597–613 (2010).

60. Mockford, E. L. North American Psocoptera (Insecta). Flora & Fauna Handbook **10**: 1–455 (1993).

61. Evans, J. W. The morphology of the head of Homoptera. Pap. Proc. Roy. Soc. Tasm. **1937**: 1–20 (1938).

62. Yoshizawa, K., Saigusa, T. Reinterpretations of clypeus and maxilla in Psocoptera, and their significance in phylogeny of Paraneoptera (Insecta: Neoptera). Acta Zool. **84**: 33–40 (2003). doi: 10.1046/j.1463-6395.2003.00127.x.

63. Grimaldi, D. A., Engel, M. S. Evolution of the Insects. Cambridge: Cambridge Univ. Press (2005).

64. Evans, J. W. The maxillary plate of Homoptera Auchenorrhyncha. J. Entomol. (A) **48**: 43–47 (1973).

65. Spangenberg, R. et al. The cephalic morphology of the Gondwanan key taxon *Hackeriella* (Coleorrhyncha, Hemiptera). Arthropod Struct. Dev. **42**: 315–337 (2013). PMID: 23583344.

66. Spangenberg, R. The evolution of head structures in Acercaria (Insecta). PhD Thesis, Friedrich-Schiller-Universität Jena, 413 pp. (2014).

67. Singh, S. Morphology of the head of Homoptera. Res. Bull. Panjab Univ. (NS) **22**: 261–316 (1971).

68. Bourgoin, T. Valeur morphologique de la lame maxillaire chez les Hemiptera; remarques phylogénétiques. Ann. Soc. Entomol. Fr. (NS) **22**: 413–422 (1986).

69. Parsons, M. C. The morphology and possible origin of the Hemipteran loral lobes. Can. J. Zool. **52**: 189–202 (1974).

70. DuPorte, E. M. The anterior tentorial arms in insects and their significance in interpreting the morphology of the cranium of cicadas. Can. J. Zool. **40**: 137–144 (1962).

71. Reyne, A. Untersuchungen über die Mundteile der Thysanopteren. Zool. Jb. Anat. **49**: 391–500 (1927).

72. Heming, B. S. Structure and function of the mouthparts in larvae of *Haplothrips verbasci* (Osborn) (Thysanoptera, Tubulifera, Phlaeothripidae). J. Morphol. **156**: 1–38 (1978).

73. Bhatti, J. S. New perspectives in the structure and taxonomy of Tubulifera. Zoology **5**: 147–176 (1998).

74. Chaudonneret, J. Les pièces buccales des insectes. Thème et variations. Edition hors-série du Bulletin Scientifique de Bourgogne: 1–256 (1992).

75. Nel, P., Azar, D., Nel, A. A new ‘primitive’ family of thrips from the Lower Cretaceous Lebanese amber (Insecta, Thysanoptera). Cretaceous Research **28**: 1033–1038 (2007).

76. Beutel, R. G., Gorb, S. N. Ultrastructure of attachment specializations of hexapods (Arthropoda): evolutionary patterns inferred from revised ordinal phylogeny. Z. Zool. Syst. Evolutionforschung **39**: 177–207 (2001).

77. Kéler, S. Über den feineren Bau der Tarsen bei Pseudomenopon rowanae Keler (Mallophaga). Beitr. Entomol. **2**: 573–582 (1952).

78. Beutel, R. G., Gorb, S. N. A revised interpretation of the evolution of attachment structures in Hexapoda with special emphasis on Mantophasmatodea. Arthropod Systematics & Phylogeny, **64**: 3–25 (2006).

79. Yoshizawa, K., Saigusa, T. Phylogenetic analysis of paraneopteran orders (Insecta: Neoptera) based on forewing base structure, with comments on monophyly of Auchenorrhyncha. Syst. Entomol. **26**: 1–13 (2001). doi: 10.1046/j.1365-3113.2001.00133.x.

80. Wheeler, W.C., Whiting, M., Wheeler, Q. D., Carpenter, J.M. The phylogeny of the extant hexapod orders. Cladistics **17**: 113–169+404 (erratum) (2001). doi: 10.1006/clad.2000.0147.

81. Dathe, H. H. Hymenoptera. In: Dathe, H. H. editor. Lehrbuch der Speziellen Zoologie, Band 1. Wirbellose Tiere, 5. Teil Insecta. Heidelberg: Spektrum: pp. 585–651 (2003).

82. Bourgoin, T. Female genitalia in Hemiptera Fulgoromorpha, morphological and phylogenetic data. Ann. Soc. Entomol. Fr. (NS) **29**: 225–244 (1993).

83. Mound, L. A., Heming, B. S., Palmer, J. M. Phylogenetic relationships between the families of recent Thysanoptera. Zool. J. Linn. Soc. **69**: 111–141 (1980). doi: 10.1111/j.1096-3642.1980.tb01934.x.

84. Yoshizawa, Y. Morphology of Psocomorpha (Psocodea: ‘Psocoptera’). Insecta Matsum. (NS) 62: 1–44 (2005).

85. Rheinwald, G. The position of *Trochiliphagus* Carriker within the Ricinidae (Insecta: Phthiraptera). Bonner zool. Beitr. **55**: 37–46 (2006).

Supporting Information

- **S1 Fig. Method of preparation of specimens and extraction of pollen grains.**
  (a) Amber cut and polished manually. (b) Polished piece heated to boiling in Canada balsam. (c) Amber polished to reach margin of bubble. (d) Surface drilled with thin pin and bubble filled with Canada balsam. (e) Curved pin piercing the abdomen to remove pollen grains from abdominal wall. (f) Drawn tip of Pasteur pipette introduced into abdomen to extract palynomorphs. (g-h) Volume renderings of segmented synchrotron radiation micro-CT scans of specimen SMNS BU-135, pollen gut contents highlighted in orange color (drawings DA).
- **S2 Fig. Head structures of *Psocorrhyncha burmitica* gen. et sp. nov., paratype NIGP161474.**
  (a) Right mandible showing molar plate. (b) Right subgena and postgena. (c) Galea and lacinia. (d) Lacinia, photomicrograph under green fluorescence. (e) Right dorso-lateral view of head. (f) General habitus. (g) base of right antenna, arrow: lateral antennifer. (h) Head, ventral view. Ga. galea; Lac. lacinia; Man. mandible; A.g. anterior part of gena; P.g. posterior part of gena; Mo. molar plate; pe. pedicel; Postgn. postgena; Sc. Scape; Subgn. Subgena. Scale bars, 0.1 mm (a, b, c, e, h), 0.2 mm (D), 1.0 mm (g).
- **S3 Fig. *Psocorrhyncha burmitica* gen. et sp. nov., allotype SMNS Bu-157.**

(a) General habitus, lateral view, arrow first abdominal segment. (b) Head, frontal view, arrows paraclypeus. (c) Wings. (d) Female genitalia, latero-ventral view. (e) Head and thorax, dorsal view, arrows ocelli. (f) Female genitalia, lateral view. Epi. epiproct; GoVIII gonocoxite VIII; GoIX gonocoxite IX; GyVIII gonapophyse VIII; GyIX gonapophyse IX; LtVIII laterotergite VIII; LtIX laterotergite IX; Pa. paraproct; T.f. trichobothrial field; TX tergite X. Scale bars, 500 µm (a, c, e), 0.1 mm (b), 200 µm (d, f).

- **S4 Fig. *Psocorrhyncha burmitica* gen. et sp. nov., paratype SMNS Bu-135.**

(a) General habitus, lateral view. (b) Head, lateral view. (c) Forewing. (d) Female genitalia. (e) Foreleg. (f) Midleg. (g) Hindleg. Scale bars, 1.0 mm (a), 200 µm (b, e, f, g), 500 µm (c).

- **S5 Fig. Morphological structures of Permopsocida.**

(a-c) *Psocorrhyncha burmitica* gen. et sp. nov., holotype NIGP161473. (A) Apical part of first flagellomere, arrow: sensilla. (b) Apical part of second flagellomere, arrow: sensilla. (c) Male genitalia. (d) Tarsi, *Archipsylla sinica* Huang *et al*., 2008, white arrows: arolia. (e) *Archipsylla sinica* Huang *et al*., 2008, Specimen NIGP161884, general habitus; A.g. anterior part of gena; P.g. posterior part of gena. Aed. aedeagus; D.e. ductus ejaculatorius; Hy hypandrium; St VIII sternite VIII; St. IX sternite IX; T. VIII tergite VIII; T. IX tergite IX. Scale bars, 0.1 mm (a, b, c, d), 1.0 mm (e).

- **S6 Fig. Morphological structures of Permopsocida.**

(a-b) *Psocorrhyncha burmitica* gen. et sp. nov., paratype NIGP161474. (a) Wing base sclerites. (b) Fore- and hind wings pterostigmata. (c) Holotype NIGP161473, detail of forewing return of ScP from C to RA. (d) *Dichentomum grande* Carpenter, 1933, Holotype MCZ 3358 forewing. BR & 2AX basiradiale and second axillary sclerite; C

costa; CuA cubitus anterior; CuP cubitus posterior; HP & Bsc humeral plate and basisubcostale plate; M median; RA radius anterior; RP radius posterior; ScP subcostal posterior. Copyrights for MCZ 3358 belong to Museum of Comparative Zoology at Harvard University. Scale bars, 0.04 mm (a), 0.1 mm (b, c), 1.0 mm (d).

- **S7 Fig. Specimen NIGP161883, *Archipsylla sinica* Huang *et al*., 2008 with possible sporangium in gut.**

  (a-b) General habitus, print and counterprint. (c-d) enigmatic structures in gut, under normal light and electron scanning microscope. Scale bars, 2.0 mm (a, b), 0.2 mm (c, d).

- **S8 Fig. Details of morphology of *Dichentomum tinctum* Tillyard, 1926.**

  (a) Specimen MCZ 3324b, head structures, arrows: maxillary palps. (b) Specimen MCZ 3347b, wings and mid leg, arrows: tarsomeres. (c) Specimen MCZ 3348, habitus, arrows indicate limits of basal flagellomeres. (d) Specimen MCZ 3331b, Head and thorax. Ga. galea; La. labrum; Man. mandible; P.g. posterior part of gena; Par.cl. paraclypeus; Pt pterostigma. Copyrights for the specimens Nos. MCZ 3324b, MCZ 3331b, MCZ 3347b and MCZ 3348 belong to Museum of Comparative Zoology at Harvard University. Scale bars, 1.0 mm (a, b, c, d).

- **S9 Fig. Morphology of Psocidiidae, Fatjanopteridae, and Hypoperlidae.**

  (a) *Stenopsocidium elongatum* Tillyard, 1935, holotype NHM In 46397, arrow: elongate mouthparts. (b) *Fatjanoptera mnemonica* Martynova, 1961, holotype PIN 1216/4, forewing. (c) *Hypoperla elegans* Martynov, 1928, holotype PIN 117/968, forewing. (d) *Hypoperla elegans*, PIN 3353/471, hind wing. (e) *Fatjanoptera mnemonica*, holotype PIN 1216/4, forewing reconstruction(drawn AN, JP). (f) '*Idelopsocus*' cf. *splendens*, PIN 1700/3298, habitus. cua-cup crossvein between CuA and CuP; CuA cubitus anterior; CuP cubitus posterior; M median; RA radius anterior;

RP radius posterior; ScP subcostal posterior. Copyrights for NHM In 46397 belong to The Natural History Museum, London. Scale bars, 1.0 mm (a), 2.0 mm (d), 5.0 mm (b, e, f).

- **S10 Fig. Morphology of hypoperlid '*Idelopsocus*' *splendens*, PU 2/129.**

  (a) Imprint, general habitus. (b) Counterimprint, general habitus. (c) Fore tarsi. (d) Head, imprint. (e) Imprint, apex of abdomen, arrow: cercus. (f) Conterimprint, apex of abdomen, arrow: cercus. La labrum; Max.palp maxillary palp. Scale bars, 1.0 mm (a, b, c), 500 µm (d, e, f).

- **S11 Fig. Phylogeny of Acercaria.**

  Most parsimonious cladogram, length = 100 steps, CI = 0.730, RI = 0.833; Bremer values indicated (drawn RG).

- **S12 Fig. Paraclypeus and gena in Hemiptera: Lachnidae and Thripida.**

  (a) Recent *Stomaphis* species, head, dorsal view. (b) *Moundthrips beatificus* Nel *et al*., 2007, holotype J2A Azar Coll., head ventro-lateral view. La. labrum; Man. mandible; A.g. anterior part of gena; P.g. posterior part of gena; Par.cl. paraclypeus; Tor. antennal torulus. Scale bars, 0.2 mm (A), 0.01 mm (B).

- **S1 Table. List of species included in Permopsocida**
- **S2 Table. List of taxa used in the phylogenetic analysis**
- **S3 Table. Characters and character states used in the phylogenetic analysis**
- **S4 Table. Data matrix of taxa and characters**
- **S5 Table. Comparison of species numbers in acercarian orders.**

S1 Table. List of species included in Permopsocida

Family	Genus	Species	Age
Archipsyllidae	*Eopsylla*	*E. sojanense* (Bekker-Migdisova, 1962)	Upper Permian
	Archipsylla	*A. primitiva* Handlirsch, 1906	Lower Jurassic
		A. sinica Haung et al., 2008	Middle Jurassic
		A. turanica Martynov, 1926	Upper Jurassic
		A. lata Vishniakova, 1976	Upper Jurassic
		A. similis Vishniakova, 1976	Upper Jurassic
	Archipsyllodes	*A. speciosus* Vishniakova, 1976	Lower Cretaceous
	Archipsyllopsis	*A. baissica* Vishniakova, 1976	Lower Cretaceous
	Psocorrhyncha	*P. burmitica* sp. nov.	Upper Cretaceous
Psocidiidae	*Dichentomum*	*D. tinctum* Tillyard, 1926	Lower Permian
		D. complexum Carpenter, 1926	Lower Permian
		D. grande Carpenter, 1933	Lower Permian
		D. latum Carpenter, 1932	Lower Permian
		D. minimum Carpenter, 1932	Lower Permian
		D. parvulum Carpenter, 1932	Lower Permian
		D. arroyo Rasnitsyn, 2004	Lower Permian
	Liassopsocus	*L. lanceolatus* Ansorge, 1996	Lower Jurassic
	Austropsocidium	*A. pincombei* Tillyard, 1935	Upper Permian
		A. stigmaticum Tillyard, 1935	Upper Permian

	Megapsocidium	*M. australe* Tillyard, 1935	Upper Permian
	Stenopsocidium	*S. elongatum* Tillyard, 1935	Upper Permian
Permopsocidae	*Permopsocus*	*P. latipennis* Tillyard, 1926	Lower Permian
	Lithopsocidium	*L. permianum* Carpenter, 1932	Lower Permian
	Orthopsocus	*O. singularis* Carpenter, 1932	Lower Permian
	Progonopsocus	*P. permianus* Tillyard, 1926	Lower Permian

Remarks

a) *Archiconiopteryx liasina* (Handlirsch, 1906) (Liassic, Dobbertin, Germany) was originally[42] included in genus *Archipsylla* Handlirsch, 1906, but later transferred to the genus *Archiconiopteryx* in Neuroptera: Coniopterygidae[43], and then revised again[44] and transferred to the sternorrhynchan family Archiconiopterygidae Ansorge, 1996.

b) *Eopsylla sojanense* was originally placed in psocidiid genus *Dichentomum*[45]; Rasnitsyn[15] proposed to remove it to the Psocidiidae because of 'possessing a complete ScP unlike the Mesozoic Archipsyllidae', which is contradictory to the reconstruction of Vishniakova[45]. This taxon should be revised.

c) ?*Dichentomum arroyo* Rasnitsyn, 2004 and ?*Dichentomum* sp., from the Carrizo Arroyo Permian[46], are only partially preserved with their basal halves of the wings missing, and are too fragmented to safely be attributed to the Permopsocida.

d) The phylogenetic position of *Dichentomum* (*Parapsocidium*) *uralicum* (Zalessky, 1937) remains ambiguous, although Carpenter[47] synonymized the genus *Parapsocidium* Zalessky, 1937 with *Dichentomum*, without clear explanation. *Parapsocidium uralicum* shares with *Dichentomum* and the Permopsocida a strong posterior angle of RA below the pterostigma, a sclerotized pterostigma, and the same pattern of branching of RP, M, and CuA in the forewing. It is likely a Permopsocida, although we do not know if it had pterostigmata on the

hind wings. Its areola postica longer than broad suggests a position near or in the Psocidiidae rather than the Permopsocidae.

e) *Liassopsocus lanceolatus* shares with the Permian Psocidiidae a vein ScP terminating into RA, but also shared with the Archipsyllidae a RA strongly angular in the pterostigma, thus its position remains uncertain even if it is a Permopsocida.

f) Following the reconstruction proposed by Tillyard[48], *Austropsocidium* Tillyard, 1935 strongly differs from Permopsocida in the absence of pterostigma in the hind wings and that RA does not form a deep curve and angle below the forewing pterostigma. The other wing venation characters (areola postica, M forked twice, RP forked) are not apomorphies of the Permopsocida. The base of M+CuA distal of wing base suggests that it is not a psocodean. As all of the body characters are unknown, it is not possible to assert it is a Permopsocida. It could belong to the stem group of the Acercaria. *Austropsocidium stigmaticum* Tillyard, 1935 is based on the distal two-thirds of a wing[48]. The form of the pterostigma with RA not exhibiting a strong posterior angle would exclude this taxon from the Permopsocida, made further complicated as the organization of the bases of M and CuA are unknown. It is probably best considered as 'Acercaria *incertae sedis*'.

g) The lack of a strong posterior angle formed below and of the pterostigma with RA would exclude *Megapsocidium* Tillyard, 1935 from the Permopsocida. Furthermore the organization of the bases of M and CuA are unknown and it should likely be placed as 'Acercaria *incertae sedis*'.

h) Tillyard's reconstruction of *Stenopsocidium elongatum* strongly differs from the original wing[48], and the forewing pterostigma fits well with those of *Psocorrhyncha* and Permopsocida in the presence of a posterior curve of RA below it and presence of a basal vein closing it. The main difference with other Permopsocida is the absence of a crossvein between RA and RP below the pterostigma, which is a rather variable character, even among modern

Psocodea (Fig. S9a). *Stenopsocidium* also shares with *Psocorrhyncha* elongate mouthparts with long mandibles and labrum; Jell[49] presented a photograph of a complete forewing from the Upper Permian of Australia that is clearly a Permopsocida owing to the shape of the pterostigma, RA, RP, M, CuA, etc. Its ScP terminates on RA closer to the pterostigma than to the base of RP, a character present in Psocidiidae.

i) Nel et al.[7] re-analysed the pattern of wing venation of *Permopsocus* and determined it to be clearly of acercarian type. Specimen number '3992a-b' depicted in a photograph in Carpenter[50], of great interest as it has an elongate prognathous head with large compound eyes and long mouthparts, similar to those of other Permopsocida, a constriction between the thorax and abdomen and a long structure corresponding to a large sclerotized spoon-like male hypandrium. Carpenter[19] determined the antenna of specimen number '3155' to have moderately long flagellomeres. The wing venation shows all diagnostic characters of Permopsocida.

j) *Lithopsocidium permianum* is based on isolated wings[19-20]. Nel et al.[7] verified its venation to be of acercarian type.

k) *Orthopsocus singularis* is based on an isolated wing[19]. Although Nel et al.[7] could not verify the pattern of venation fits with Acercaria, its great similarity to that of *Permopsocus* strongly supports an attribution to the same group.

S2 Table. List of taxa used in the phylogenetic analysis

Outgroups:

Blattodea: *Periplaneta americana* (Linnaeus, 1758) (extant)

Plecoptera: *Eusthenia costalis* Banks, 1913 (extant)

Zoraptera: *Zorotypus caudelli* Karny, 1927 (extant)

Holometabola: *Xyela julii* (Brébisson, 1818) (extant)

Ingroups:

Hypoperlidae:

Hypoperla elegans Martynov, 1928 (based on reexamined photographs of the type wings) (Permian)

Idelopsocus splendens (Zalessky, 1948) (based on revision of specimens PIN 1700/3298 and PU 2/129) (Permian)

Permopsocida:

Archipsylla sinica Huang et al, 2008 (Middle Jurassic)

Dichentomum tinctum Tillyard, 1926 (based on present revision) (Permian)

Permopsocus latipennis Tillyard, 1926 (Permian)

Psocorrhyncha burmitica gen. nov., sp. nov. (Cretaceous)

Psocodea:

Burmacompsocus perreaui Nel & Waller, 2007 (Compsocidae) (Cretaceous)

Libanomphientomum nudus Choufani et al., 2011 (Amphientomidae) (Cretaceous)

Thripida:

Moundthrips beatificus Nel et al., 2007 (Cretaceous)

Thrips tabaci Lindeman, 1889 (extant)

Hemiptera:

Archescytina sp. (Archescytinidae, supposed most basal clade of Hemiptera, specimen with body preserved)

Southia opposita (F., 1803) (Fulgoromorpha: Kinnaridae) (extant)

S3 Table. Characters and character states used in the phylogenetic analysis

1. Head: (0) not opisthognathous; (1) opisthognathous, orientated obliquely, with mouthparts pointed backward (Palaeozoic and some Mesozoic Thripida have a prognathous or hypognathous head, while the head is opisthognathous in modern Thysanoptera[51]. The opisthognathy cannot be considered a synapomorphy of Thripida and Hemiptera. The Permopsocida have hypognathous heads) (state 0 for *Psocorrhyncha*)
2. Sclerotized ring at base of first antennal flagellomere, inside pedicel: (0) absent; (1) present (a character of Hemiptera and modern Thysanoptera[52-53]) (state 0 for *Psocorrhyncha*)
3. Rupturing mechanism at base of antennal flagellum: (0) absent; (1) present (a character of Psocodea[11,53]) (state 0 for *Psocorrhyncha*)
4. Flagellomeres annulated with cuticular sculpture: (0) present; (1) absent (annulation is present in Psocodea: Troctomorpha, in some Thripida, Hemiptera: Aphidoidea, Isoptera, Mantophasmatodea, Ephemeroptera, and Plecoptera) (state 0 for *Psocorrhyncha*)
5. Insertion of scape on head capsule by a dicondylar articulation (acute lateral antennifer and weaker median articulation point on head capsule): (0) present; (1) absent (a dicondylar articulation occurs in modern Thysanoptera[53], Orthoptera, Phasmatodea, and Thysanura[54-56], while other insects have a ball-and-socket joint[56]. Psocodea have a single condyle or no condyle[53,57]. While Heming[53] considered the dicondylar articulation as derived in Thysanoptera, its presence in Thysanura, Orthoptera, and Phasmatodea suggests it could be a plesiomorphy for the Insecta) (state 0 for *Psocorrhyncha*)
6. Position of anterior tentorial pits: (0) frontal side of head; (1) shifted dorsally (the anterior tentorial pits are absent in Anoplura and Rhynchophthirina, not considered

here; they are shifted dorsally in Hemiptera and modern Thysanoptera, but not in Palaeozoic and Mesozoic Thripida[11,58]) (state 0 for *Psocorrhyncha*)

7. Dorsal part of head with a sub-horizontal posterior part and a subvertical anterior part bearing the ocelli: (0) no (Psocodea, Thripida); (1) yes (state '0' occurs in outgroups, Psocodea and Thripida, state '1' occurs in Hemiptera: Fulgoromorpha[58-60]) (state 1 for *Psocorrhyncha*)
8. Ocell-ocular distance < inter-ocellular distance: (0) no ; (1) yes (state '1' occurs in those Hemiptera with a broad clypeo-frons[61]) (state 1 for *Psocorrhyncha*)
9. Clypeus divided by a furrow into ante- and postclypeus: (0) no; (1) yes (state '1' in some Hemiptera (e.g. Cicadoidea) but not all (e.g Aphidoidea), Thripida, and some Psocodea[62]) (state 1 for *Psocorrhyncha*)
10. Postclypeus: (0) not very large and bulbous; (1) large, bulbous, with large cibarial dilator muscles (this character state is currently assigned to the Acercaria[62-63]), but the postclypeus is not as large and bulbous in the Palaeozoic or Mesozoic Thripida nec. Thysanoptera as in Psocodea, extant Thysanoptera, and Hemiptera. Therefore the large postclypeus of Psocodea, extant Thysanoptera and Hemiptera is certainly a convergence. In Psocodea, the frons is well separated from the postclypeus, unlike in modern Thysanoptera, and probably Hemiptera, although terminology for the latter clade is controversial[59,62,64]) (state 0 for *Psocorrhyncha*)
11. Paraclypeal lobes: (0) not separated and not distinct from median part of (ante)-clypeus; (1) separated and distinct from median part of (ante)-clypeus (Presence of two relatively sclerotized paraclypeal lobes[65] is an apomorphic character present in recent and fossil Thripida[58]. Hemiptera also have sclerotized sclerites in the same position as the paraclypeal lobes of Thripida and of *Psocorrhyncha*. Some authors confused the mandibular plate (lora) for paraclypeus (see [59] for summary of diverse

opinions). Spangenberg et al.[65] and Spangenberg[66] confirmed the opinion of Singh[67] about the fact that the paraclypeus of Hemiptera is a structure different from the mandibular plate. In Coleorrhyncha[65], the paraclypeal lobes are visible in dorsal view, placed laterally to the anterior part of the anteclypeus while mandibular plates are visible only in lateral view; these structures are fused but separated internally by a 'distinct cresent-shaped apodeme'[65]. In the aphidoidean *Stomaphis*, the paraclypeal lobes are very broad structures (Fig. S13a). The clypeus of the Psocodea, Hypoperlidae, and other Insecta is not clearly differentiated into paraclypeal lobes and a median part. This character constitutes a potential synapomorphy of a clade comprising Permopsocida, Thripida, and Hemiptera. *Stenopsocidium elongatum* could also have two sclerotized paraclypeal lobes (Fig. S9a). Also *Dichentomum tinctum* has two small rounded sclerites at the base of the labrum corresponding to paraclypeal lobes, see Fig. S8a) (state 1 for *Psocorrhyncha*)

12. Median part of (ante)-clypeus: (0) not membraneous; (1) membraneous (state '1' in Thripida[58]; a potential synapomorphy of Permopsocida and Thripida, modified in Hemiptera in relation to the hyper-development of the clypeus) (state 1 for *Psocorrhyncha*)
13. Labrum: (0) not elongate, less than two times longer than broad; (1) elongate, two times longer than broad or more (state '1' in Hemiptera and Thripida) (state 1 for *Psocorrhyncha*)
14. Left mandible: (0) not stylet-like; (1) stylet-like (state '1' in Hemiptera and Thripida) (state 0 for *Psocorrhyncha*)
15. Right mandible: (0) not stylet-like; (1) stylet-like (state '1' in Hemiptera; the elongate mandibles with a broad base together with the elongate labrum in *Psocorrhyncha* fits well with the "Hypothetical scheme of transformations of chewing mandibles into

stylets" proposed by Emeljanov[30], placing *Psocorrhyncha* between his steps "(1)" (psocodean state) and "(2)". Nevertheless the mandibles of Permopsocida are clearly plesiomorphic compared to the stylet-like mandibles of Thripida and Hemiptera) (state 0 for *Psocorrhyncha*)

16. Right mandible: (0) present; (1) absent, mouthcone asymmetrical (state '1' in Thripida[51]) (state 0 for *Psocorrhyncha*)
17. Maxillary lacinia: (0) in direct contact with stipes; (1) not in direct contact with stipes, probably independently movable (putative apomorphy of Acercaria[11], there is an intermediate structure between the lacinia and the stipes in modern Thysanoptera) (state 1 for *Psocorrhyncha*)
18. Lacinia: (0) with at least one subapical tooth; (1) without any subapical tooth (in Orthoptera, Phasmatodea, Plecoptera, and Psocodea, the lacinia has at least one strong subapical tooth, except in few Caeciliidae, while in Thripida and Hemiptera there is only an acute apical tooth) (state 1 for *Psocorrhyncha*)
19. Lacinia: (0) distally broad; (1) stylet-like distally (a broadened distal part of lacinia is a plesiomorphic character state present in Psocodea, compared to the acute and thin lacinia of Thripida and Hemiptera[57]; note the eucinetid beetle *Jentozkus plaumanni* has stylet-like lacinia, plus galea) (state 0 for *Psocorrhyncha*)
20. Lacinia: (0) not elongate; (1) elongate (state '1' in Acercaria, but elongate lacinia cannot be considered as a strict synapomorphy of Acercaria because elongate lacinia occur frequently when the head and mouthparts are elongate (e.g., the mecopteran genus *Panorpodes*) (state 1 for *Psocorrhyncha*)
21. Cardo and stipes: (0) separated by a furrow; (1) fused (The cardo and stipes separated by a furrow is a plesiomorphy relative to their fusion in Psocodea[11,62]) (state 0 for *Psocorrhyncha*)

22. Gena: (0) not subdivided into two parts, (1) subdivided into two parts by a strong furrow (The gena is subdivided into two parts by a strong furrow in *Psocorrhyncha*. Such a subdivision of the gena is absent in Psocodea and the Hypoperlidae, but visible in *Dichentomum*. There is a controversy about the origin of the maxillary lobe of Hemiptera of genal origin[68-69], versus of appendicular origin (maxilla)[64,66]. Duporte[70] proposed that the maxillary plate could be of composite origin, due to the fusion of cardo and stipes, and that both latter in turn are fused with the genae and postgenae. Presence of a posterior lobe of gena in *Psocorrhyncha* would support the hypothesis of Bourgoin[68] because this taxon has a 'normal' maxilla not fused with the gena, and in many Hemiptera there is continuity without any maxillary suture between the posterior part of the gena and the maxillary plate. Nevertheless the problem will be really solved using the tools of the genetic of the development. The anterior part of the gena is currently called lora (for non-heteropteran Hemiptera), or mandibular plates (for Heteroptera)[65,68]. The mid Jurassic Permopsocida appear to also have a subdivision of the gena (Fig. S5e). The Thripida have also a gena subdivided into a long mandibular plate in lateral position in front of the base of the antenna plus a posterior part below the eye (visible in the Cretaceous thripidan *Moundthrips* (Fig. S13b), and present in the early nymphs of modern *Heliothrips* or *Haplothrips*[71-72]. A genal fissure is also present in the modern Tubulifera[73]. The anterior mandibular plate closes the mouthcone laterally in *Moundthrips* (Fig. S13b), but it appears fused with the maxilla in modern Thysanoptera. Emeljanov[30] proposed hypothetical stages of transformation from the 'psocodean' head to the 'hemipteran' one, with two structures progressively appearing and developing and corresponding to the mandibular and maybe the maxillary lobes, but he misplaced these structures anteriorly to the gena) (state 1 for *Psocorrhyncha*)

23. Maxillary palp: (0) five-segmented; (1) four-segmented, (2) less than four-segmented (Hemiptera and Thripida have state ‘2’) (state 1 for *Psocorrhyncha*)

24. Last maxillary palpomere: (0) inserted normally on penultimate; (1) inserted apically on penultimate, penultimate cut obliquely at its apex (*Psocorrhyncha* unique apomorphy)

25. Last maxillary palpomere: (0) without broad flat sensillar zone; (1) with broad flat sensillar zone (*Psocorrhyncha* unique apomorphy, unknown in other Permopsocida)

26. Mentum: (0) not elongated; (1) elongated (state ‘0’ in Psocodea and Hypoperlidae, the mentum are not elongate; state ‘1’ in Hemiptera and Thripida[74] (state 1 for *Psocorrhyncha*)

27. Labial palps: (0) with more than two segments; (1) absent or strongly reduced (Presence of three-segmented labial palps in *Psocorrhyncha* is plesiomorphic. Psocodea and Thripida have labial palps one- or two-segmented, while they are lost in Hemiptera) (state 0 for *Psocorrhyncha*)

28. Hypopharynx: (0) not expanded posteriorly; (1) expanded posteriorly (state ‘0’ in Psocodea, state ‘1’ in Hemiptera, modern Thysanoptera, and at least in *Moundthrips* among Mesozoic Thripida[30,75]) (state unknown for *Psocorrhyncha*)

29. Cibarial water-vapour uptake apparatus: (0) absent; (1) present (state ‘1’ in Psocodea[11]) (state unknown for *Psocorrhyncha*)

30. Pearman's organ on hind coxa: (0) absent; (1) present (state ‘1’ Psocoptera excl. Liposcelidae) (state unknown for *Psocorrhyncha*)

31. Number of tarsomeres (multistate): (0) five; (1) four; (2) three or less (five-segmented tarsi in the ground plan of Pterygota; three-segmented tarsi in the ground plan of Plecoptera, state ‘2’ in Zoraptera, Psocodea, Thripida, and Hemiptera; five-segmented tarsi in Hypoperlidae, four-segmented tarsi in Permopsocida; it is likely that reduction

in the number of tarsomeres occurred convergently in Zoraptera, Psocodea, and the clade Thripida + Hemiptera) (state 1 for *Psocorrhyncha*)

32. Paired tarsal plantulae: (0) present; (1) absent (state '1' is a character of Eumetabola = Acercaria + Holometabola; Beutel & Gorb[76] indicated the presence of 'euplantulae' in some Mallophaga, but these are unpaired structure[77], probably non homologous[78] to the euplantulae of the polyneoptera) (state 1 for *Psocorrhyncha*)
33. Claws: (0) not reduced in adult; (1) reduced in adult (state '1' in fossil and modern Thripida) (state 0 for *Psocorrhyncha*)
34. Arolium: (0) broad and fleshy; (1) arolium broad but retractile; (2) arolium reduced, only a pulvillus inserted at base of claw (state '0' in *Psocorrhyncha*, Xylelidae, many Polyneoptera, Hemiptera; state '1' in Thripida[58]; state '2' in Psocodea[11])
35. In wing articulation, humeral plate (HP) and basisubcostale (BSc): (0) separated; (1) united (state '1' apomorphy of Acercaria[79]) (state 1 for *Psocorrhyncha*)
36. In wing articulation, BSc and second axillary sclerite (2Ax): (0) separated; (1) fused (state '1' in Hemiptera[79]; state unknown in Thripida) (state 0 for *Psocorrhyncha*)
37. Fringe on posterior edge of wing: (0) absent; (1) present. (state '1' in Thripida[7]) (state 0 for *Psocorrhyncha*)
38. Forewings: (0) not more sclerotized than hind wings; (1) at least slightly more sclerotized than hind wings (state '1' in some modern Hemiptera[8]) (state 0 for *Psocorrhyncha*)
39. Wings: (0) hind wings not much smaller than forewings; (1) hind wings much smaller than forewings (state '1' in Psocodea but also in Hemiptera: Aphidoidea[8]) (state 0 for *Psocorrhyncha*)
40. A common stem R+M+CuA: (0) absent; (1) present (state '1' convergently present in Archaeorthoptera and Acercaria[7,23] (state 1 for *Psocorrhyncha*)

41. M (plus CuA if fused basally with radius) separates from R: (0) well distal of wing base; (1) very close to wing base (state '1' is proper to the Psocodea, fossil and modern, except some Troctomorpha[7]) (state 0 for *Psocorrhyncha*)
42. A neutral crossvein cua-cup between concave CuP and convex CuA, weaker than CuA: (0) absent; (1) present (state '1' in Acercaria[7]) (state 1 for *Psocorrhyncha*)
43. Radial stem at point of re-emergence of CuA and M: (0) not displaying a pronounced posterior angle; (1) displaying a strong posterior angle (Such an angle appears to be present in the hemipteran ground plan, as it can be observed in Archescytinidae and many Fulgoromorpha, but not in Psocodea or Thripida) (state 1 for *Psocorrhyncha*)
44. Areola postica: (0) absent; (1) present, longer than high; (2) present, higher than long (States '1' or '2' in Acercaria, CuA-fork is reduced in Thripida and few Psocodea) (state 2 for *Psocorrhyncha*)
45. Vein M: (0) forked into many branches; (1) forked twice into four branches M1-M2 and M3-M4; (2) forked into three pectinate branches (hemipteran ground plan?); (3) only forked once into two branches or less (State '1' in Permopsocida; the three states '1', '2', and '3' are present among various taxa in Psocodea and Hemiptera; state '3' in Thripida)
46. RP: (0) forked; (1) unforked (State '1' in the majority of Hemiptera, but not all) (state 0 for *Psocorrhyncha*)
47. Pterostigma in forewing: (0) absent; (1) present but not limited by costal wing margin and vein RA, more sclerotized than rest of wing; (2) present, limited by costal wing margin and vein RA, more sclerotized than rest of wing (State '2' in psocodean ground plan; Thripida have no pterostigmata; Hemiptera have forewing pterostigmata in their ground plan, present in Archescytinidae and some Fulgoromorpha, Aphididae, etc.) (state 2 for *Psocorrhyncha*)

48. Pterostigma in hind wing: (0) absent; (1) present but not limited by costal wing margin and vein RA, more sclerotized than rest of wing; (2) limited by costal wing margin and a deep posterior curve of vein RA, more sclerotized than rest of wing (State 2 for Permopsocida, autapomorphy; similar hind wing pterostigmata are also present in holometabolous Raphidioptera; Hemiptera Archescytinidae also have pterostigmata in their fore- and hind wings, but of different shape)

49. Forewing ScP: (0) parallel to radius and fusing with it far from wing base; (1) fused with costa near wing base but re-emerging distally to end in radius; (2) fused with costa near wing base and not re-emerging (homoplastic character states as the two situations '0' and '2' can occur in the same family of Psocodea, and in different taxa of Permopsocida; state '1' occurs also in the psocodean family Lepidopsocidae but with ScP only fused for a short length with costa)

50. Anal veins in fore wings: (0) more than two free anal veins; (1) two free anal veins or less (state '1' in Acercaria) (State 1 for *Psocorrhyncha*)

51. Coupling of fore- and hind wings with stigmapophysis in rest (a blunt chitinous projection at base of pterostigma of forewing): (0) absent; (1) present (State '1' in winged Psocodea) (state 0 for *Psocorrhyncha*)

52. Jugal 'bar': (0) absent; (1) present (State '1' in Eumetabola; definitely not present in Zoraptera according to Grimaldi & Engel[63] and Friedemann et al.[11]; contra Wheeler et al.[80]); not discernable in any of the studied fossils)

53. Abdominal sternite 1: (0) present and fully developed; (1) reduced or absent (State '0' in Zoraptera; state '1' in modern Acercaria, except Thysanoptera; Friedemann et al.[11]) (state 1 for *Psocorrhyncha*)

54. Abdominal segment I: (0) not very narrow and reduced; (1) very narrow and reduced (Character state '1' present in all Permopsocida; the hypoperlid *Idelopsocus* has a

narrow segment I but less narrow than in Permopsocida; nevertheless some Burmese amber specimens and extant Psocodea (e.g. *Lachesilla*) have a similar constriction, thus this character is subject to homoplasy in Acercaria)

55. Female with reduced abdominal tergites IX and X (thripidan type): (0) no; (1) yes (state '1' in Thripida[58]) (state 0 for *Psocorrhyncha*)

56. Cerci: (0) long and multi-segmented; (1) short and one-segmented; (2) absent (State '1' in Zoraptera; state '2' in Acercaria, except in Hypoperlidae; in Hymenoptera there are 'cerci' but it is unclear if they belong to the 10th or the 11th segment[81]) (state 2 for *Psocorrhyncha*)

57. Ovipositor: (0) present and well developed; (1) reduced, of psocodean type (State '1' Psocodea; state '0' in the ground plan of Thripida, a character described by Bourgoin[82]. The female anal appendages of *Psocorrhyncha* are similar to those of Hemiptera: Fulgoromorpha of raking type[82], viz. in the presence of gonapophyses VIII with a raking structure, gonapophyses IX weaker and less sclerotized and broad weakly sclerotized gonoplacs. These anal appendages do not correspond to female anal appendages of thripidan type[58] because *Psocorrhyncha* has reduced tergites IX and X. *Psocorrhyncha* differs from those of the female Psocodea in the strong gonapophyses VIII with raking apparatus[60])

58. Female gonangulum: (0) not fused with tergum IX; (1) fused with tergum IX (State '1' in Acercaria; after Friedemann et al.[11], 'The gonangulum is fused with tergum IX in Acercaria and Odonata', and 'the situation is unknown for Enicocephalomorpha, Dipsocoromorpha, and Phthiraptera') (state unknown for *Psocorrhyncha*).

59. Gonostyli: (0) present; (1) absent, lost (state '1' in Acercaria, Zoraptera, Embioptera) (state 1 for *Psocorrhyncha*)

60. Male anal appendages more sclerotized, especially with large and strongly sclerotized spoon-like hypandrium: (0) yes; (1) no (State '1' in modern Psocodea[60]. The male anal appendages of *Psocorrhyncha* are more sclerotized than in modern Psocodea, especially in the presence of a large and strongly sclerotized spoon-like hypandrium) (state 0 for *Psocorrhyncha*)
61. Abdominal ganglia: (0) more than two separate ganglia; (1) two separate ganglia; (2) one single ganglionic mass. Two separate abdominal ganglionic complexes are found in Zoraptera. A single ganglionic mass is a possible autapomorphy of Acercaria[11].
62. Lateral hypopharyngeal arm (0) present; (1) absent. The lateral hypopharyngeal arm is absent in Psocodea and Zoraptera. It is present in Thysanoptera, Auchenorrhyncha, Aphidoidea, Psylloidea, Pentatomomorpha, Enicocephalomorpha, Dipsocoromorpha, and Coleorrhyncha. The situation is unknown for Aleyrodidae, and Coccoidea[11].

Remark. Grimaldi and Engel[63] proposed that the presence of abdominal trichobothria in winged forms is a synapomorphy of Acercaria. The most 'basal' extant Thysanoptera (Merothripidae, some Aeolothripidae) have a pair of trichobothria on the tergum X[83]. The Psocodea have a trichobothrial field on the paraprocts[84], supposedly corresponding to a 'reduced cercus' of the segment XI. *Psocorrhyncha* has the same structure, at least in the female allotype. Hemiptera have no such trichobothrial field on their reduced paraprocts, and Thripida have no visible paraproct (as a remnant of segment XI). Many Hemiptera have pairs of trichobothria on several abdominal sternites. Therefore, there is no clear reason to consider diverse abdominal trichobothria are homologous between Psocodea, Hemiptera, and Thripida. Because of this ambiguity we prefered to not include the trichobothrial character proposed by Grimaldi and Engel[63] in our matrix.

S4 Table. Data matrix of taxa and characters

Taxa/Characters	1	2	3	4	5	6	7	8	9	10	11	12	13	14	15	16	17	18	19	20	21	22	23	24	25	26	27	28	29	30	31
Eusthenia	0	0	0	0	1	0	0	0	0	0	0	0	0	0	0	0	0	0	0	0	0	0	0	0	0	0	0	0	0	0	2
Zorotypus	0	0	0	1	1	0	0	0	1	0	0	0	0	0	0	0	0	0	0	0	0	0	0	0	0	0	0	0	0	0	2
Xyela	0	0	0	1	1	0	0	0	0	0	0	0	0	0	0	0	0	0	0	0	0	0	0	0	0	0	0	0	0	0	0
Hypoperla	?	?	?	?	?	?	?	?	?	?	?	?	?	?	?	?	?	?	?	?	?	?	?	?	?	?	?	?	?	?	?
Idelopsocus	0	?	?	0	?	0	0	?	0	0	0	?	0	0	0	0	?	?	0	1	?	0	0	0	?	0	0	?	?	?	0
Psocorrhyncha	0	0	0	0	0	0	1	1	1	0	1	1	1	0	0	0	1	1	0	1	0	1	1	1	1	1	0	?	?	?	1
Archipsylla	0	?	?	0	?	0	?	1	1	0	?	?	1	0	0	0	1	1	0	1	?	?	1	?	1	?	0	?	?	?	1
Dichentomum	0	?	?	0	?	0	1	1	1	0	1	?	1	0	0	0	?	?	0	1	?	1	1	?	?	?	0	?	?	?	1
Permopsocus	0	?	?	?	?	0	1	?	?	?	?	?	1	0	0	0	?	?	?	?	?	?	?	?	?	?	?	?	?	?	1
Burmacompsocus	0	0	1	0	1	0	0	0	1	1	0	0	0	0	0	0	1	0	0	1	1	0	1	0	0	0	1	0	1	1	2
Libanomphientomum	0	0	1	0	1	0	0	0	1	1	0	0	0	0	0	0	1	0	0	1	1	0	1	0	0	0	1	0	1	1	2
Thrips	1	1	0	0	0	1	0	0	1	1	1	1	1	1	0	1	1	1	1	1	0	1	2	0	0	1	1	1	0	0	2
Moundthrips	0	?	?	?	?	0	0	0	1	0	1	1	1	1	0	1	1	1	1	1	0	1	2	0	0	1	1	1	0	0	2
Archescytina	1	?	?	?	?	?	?	?	1	1	1	0	1	1	1	0	1	1	1	1	?	1	?	0	0	1	1	?	?	?	2
Southia	1	1	0	?	?	1	1	0	1	1	1	0	1	1	1	0	1	1	1	1	0	1	2	?	?	1	1	1	0	0	2
Periplaneta	0	0	0	0	1	0	0	0	1	0	0	0	0	0	0	0	0	0	0	0	0	0	0	0	0	0	0	0	0	0	0

S4 Table. Data matrix of taxa and characters (continue)

Taxa/Characters	32	33	34	35	36	37	38	39	40	41	42	43	44	45	46	47	48	49	50	51	52	53	54	55	56	57	58	59	60
Eusthenia	0	0	0	0	0	0	0	0	0	0	0	0	0	0	0	0	0	0	0	0	0	0	0	0	0	0	0	0	0
Zorotypus	1	0	2	0	0	0	0	0	0	0	0	0	1	2	1	0	0	0	1	0	0	0	0	0	1	1	?	1	1
Xyela	1	0	0	0	0	0	0	0	0	0	0	0	0	3	1	2	0	0	1	0	1	0	0	0	?	0	0	0	0
Hypoperla	?	?	?	?	?	0	0	0	1	0	1	0	1	0	0	1	1	0	1	?	?	?	?	?	?	?	?	?	?
Idelopsocus	1	0	0	?	?	0	0	0	1	0	1	0	1	1	0	1	1	0	1	?	?	?	0	0	1	0	?	?	?
Psocorrhyncha	1	0	0	1	0	0	0	0	1	0	1	1	1	1	0	2	2	1	1	0	?	1	1	0	2	0	?	1	0
Archipsylla	1	0	0	?	?	0	0	0	1	0	1	1	1	1	0	2	2	1	1	0	?	?	1	0	2	0	?	?	?
Dichentomum	1	0	?	?	?	0	0	0	1	0	1	1	1	1	0	2	2	0	1	?	?	?	1	0	2	0	?	?	?
Permopsocus	?	?	?	?	?	0	0	0	1	0	1	1	2	1	0	2	2	0	1	?	?	?	1	?	2	?	?	?	0
Burmacompsocus	1	0	2	1	0	0	0	1	1	1	1	0	2	2	0	2	0	0	1	1	1	1	0	0	2	1	1	1	1
Libanomphientomum	1	0	2	1	0	0	0	1	1	1	1	0	2	2	0	2	0	0	1	1	1	1	0	0	2	1	1	1	1
Thrips	1	1	1	1	?	1	0	0	1	0	1	0	0	3	1	0	0	2	1	0	0	1	0	1	2	0	1	1	0
Moundthrips	1	1	1	?	?	1	0	0	1	0	1	0	0	3	1	0	0	2	1	0	0	1	0	1	2	0	1	1	0
Archescytina	?	?	?	?	?	0	0	0	1	0	1	0	1	2	1	2	0	2	1	0	?	?	0	0	2	0	?	?	?
Southia	1	0	0	1	1	0	1	0	1	0	1	0	1	2	1	2	0	2	1	0	1	1	0	0	2	0	1	1	0
Periplaneta	0	0	0	0	0	0	1	0	0	?	0	0	0	0	0	0	0	?	0	0	0	0	0	0	0	?	0	0	0

S5 Table. Comparison of species numbers in acercarian orders.

Remark. Within Psocodea-Phthiraptera sucking-piercing mouthparts evolved at least three times in convergence: in Anoplura and in the two humming bird parasites *Trochiloectes* and *Ricinus* (= *Trochiliphagus*) *jimenezi* (Amblycera)[85].

**Order**	**Stratigraphic range**	**Feeding mode**	**Species number**
Hypoperlidae	307 mya – 254 mya	chewing	13
Psocodea	315(–307) mya – Recent	chewing or sucking-piercing	11.000
Permopsocida	290(–283) mya – 99 mya	chiseling	25
Thripida (incl. Thysanoptera)	323(–315) mya – Recent	chiseling	6.000
Hemiptera	315(–307) mya – Recent	sucking-piercing	82.000

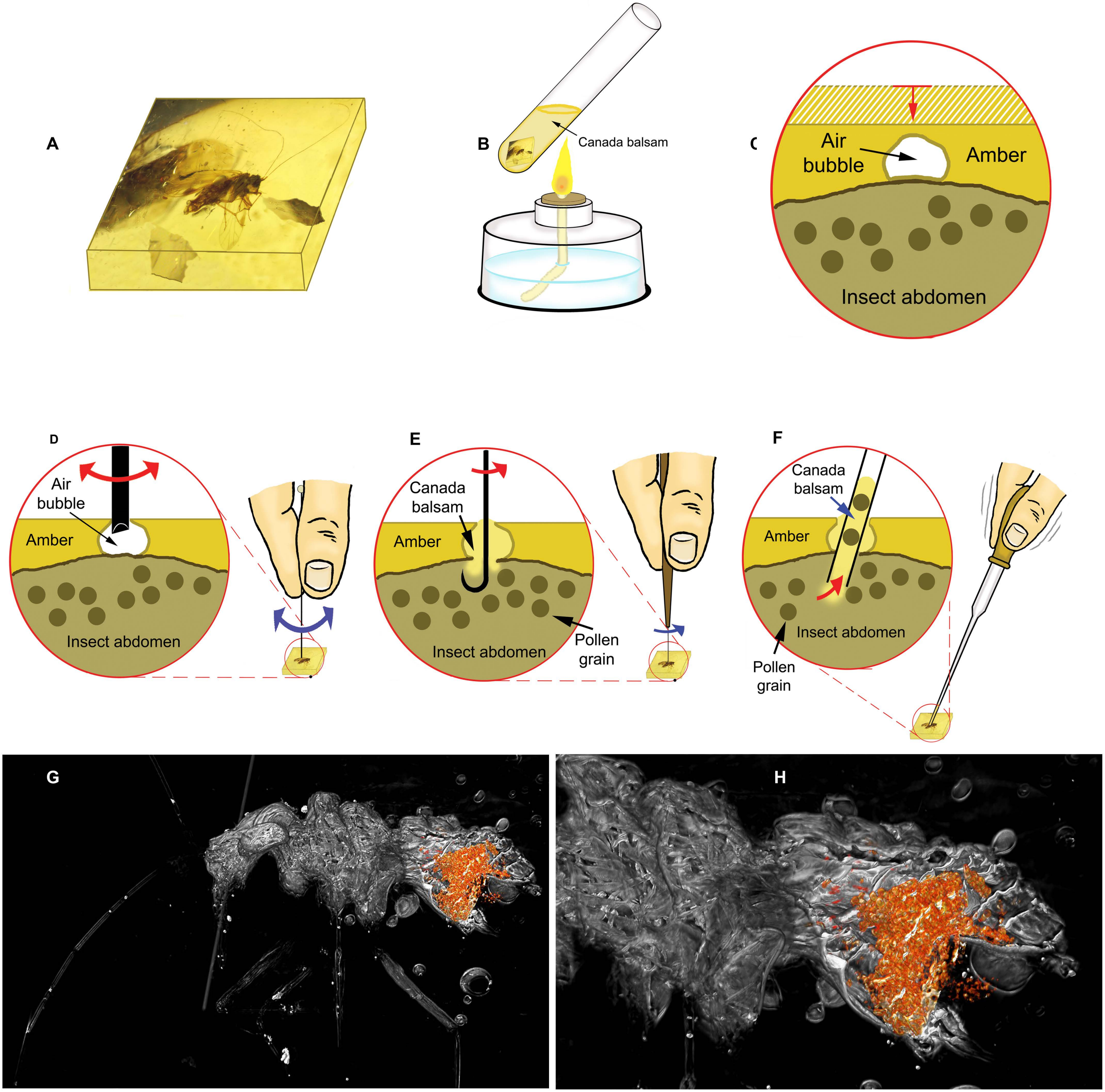

A
B
Canada balsam
C
Air bubble
Amber
Insect abdomen
D
Air bubble
Amber
Insect abdomen
E
Canada balsam
Amber
Insect abdomen
Pollen grain
F
Canada balsam
Amber
Insect abdomen
Pollen grain
G
H

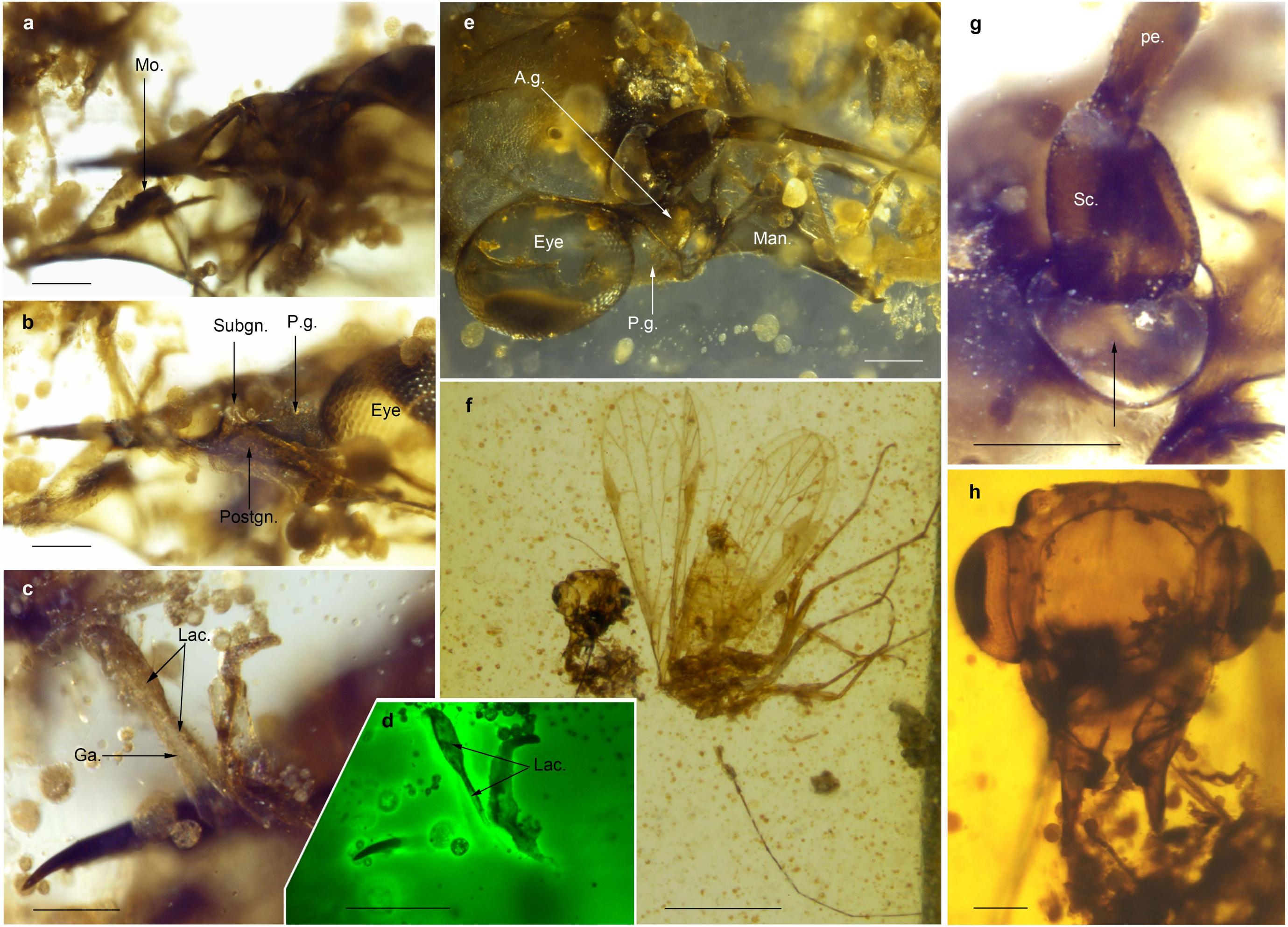
a
Mo.
b
Subgn.
P.g.
Eye
Postgn.
c
Lac.
Ga.
d
Lac.
e
A.g.
Eye
Man.
P.g.
f
g
pe.
Sc.
h

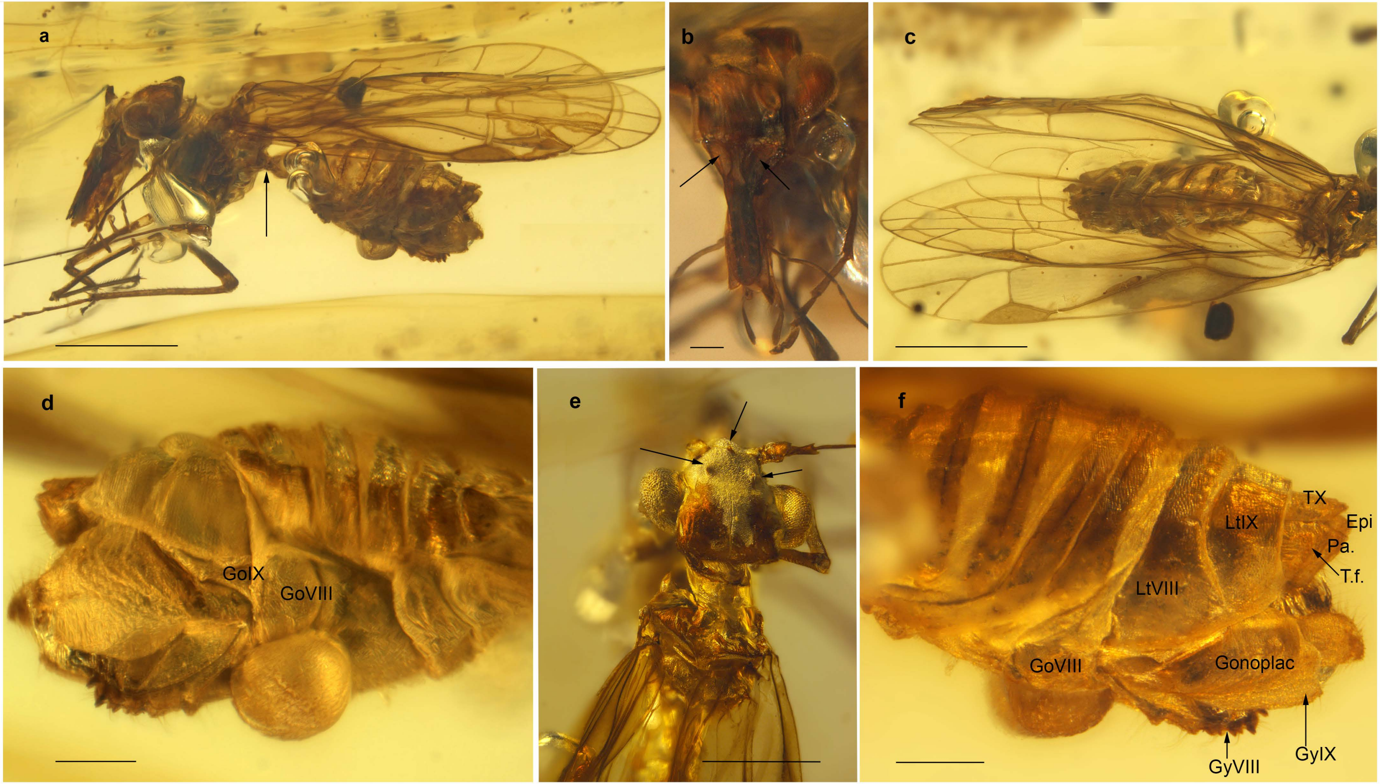
a
b
c
d
GoIX
GoVIII
e
f
TX
Epi
LtIX
Pa.
T.f.
LtVIII
GoVIII
Gonoplac
GyVIII
GyIX

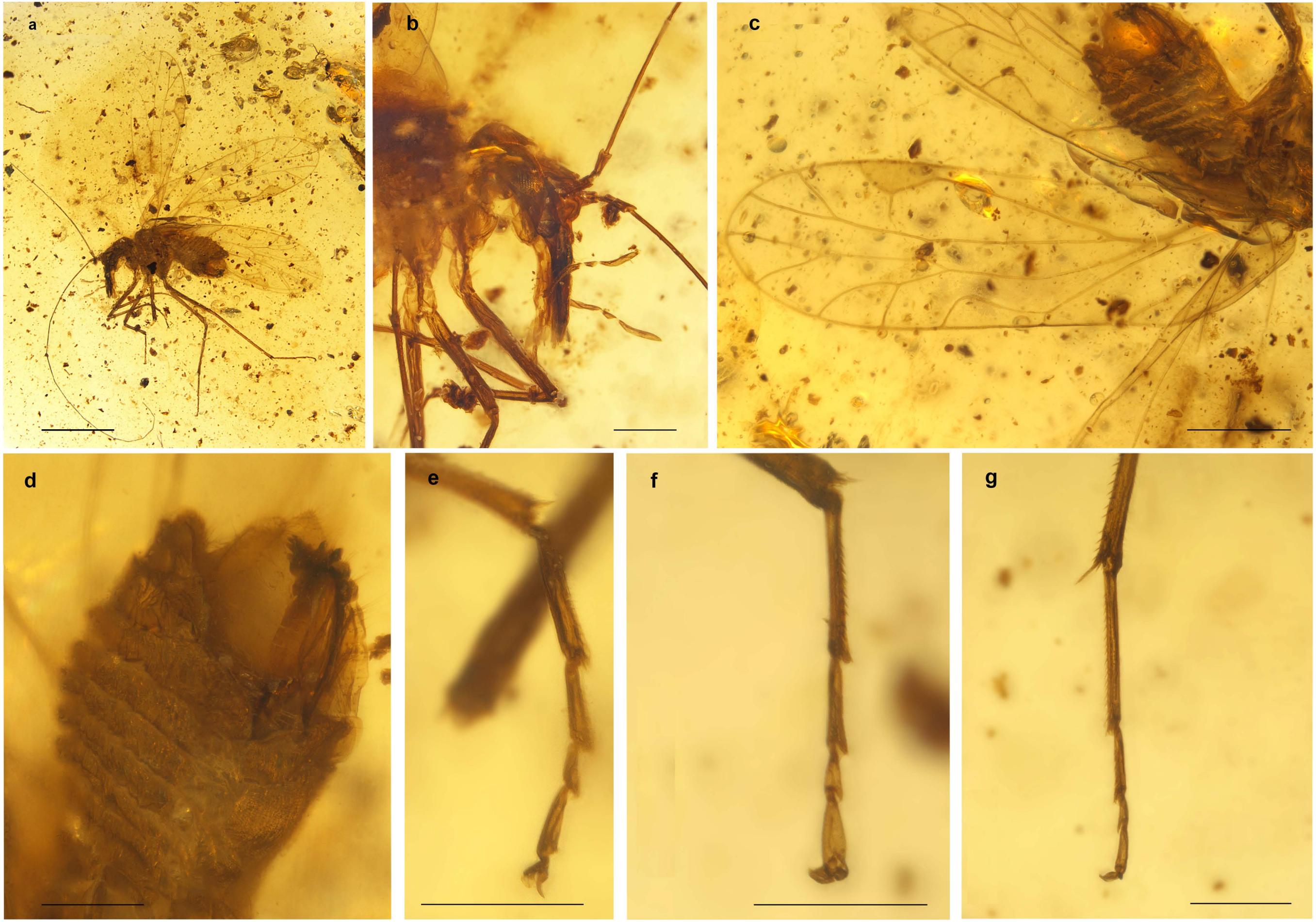
a
b
c
d
e
f
g

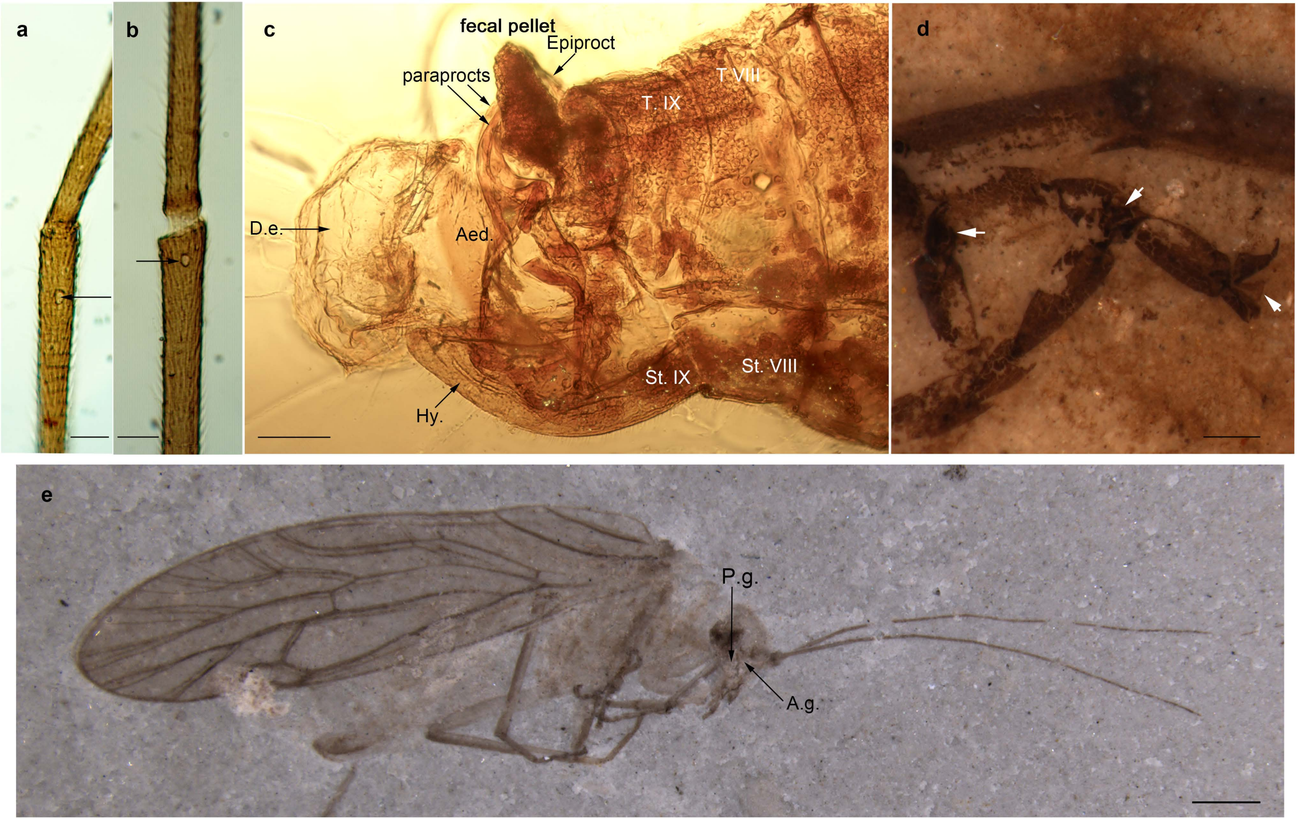

a
b
c
fecal pellet
Epiproct
paraprocts
T VIII
T. IX
D.e.
Aed.
Hy.
St. IX
St. VIII
d
e
P.g.
A.g.

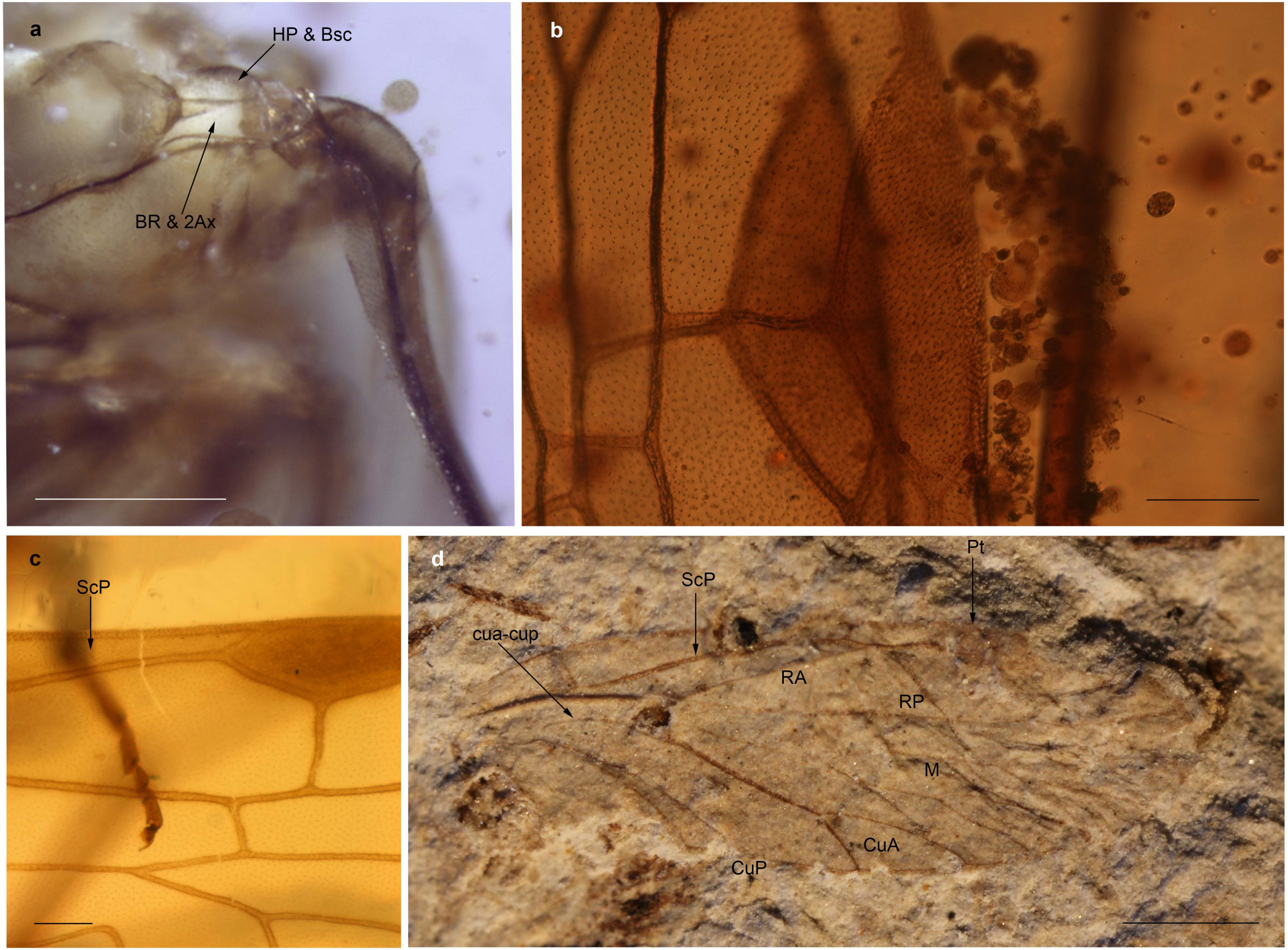
a
HP & Bsc
BR & 2Ax
b
c
ScP
d
Pt
ScP
cua-cup
RA
RP
M
CuA
CuP

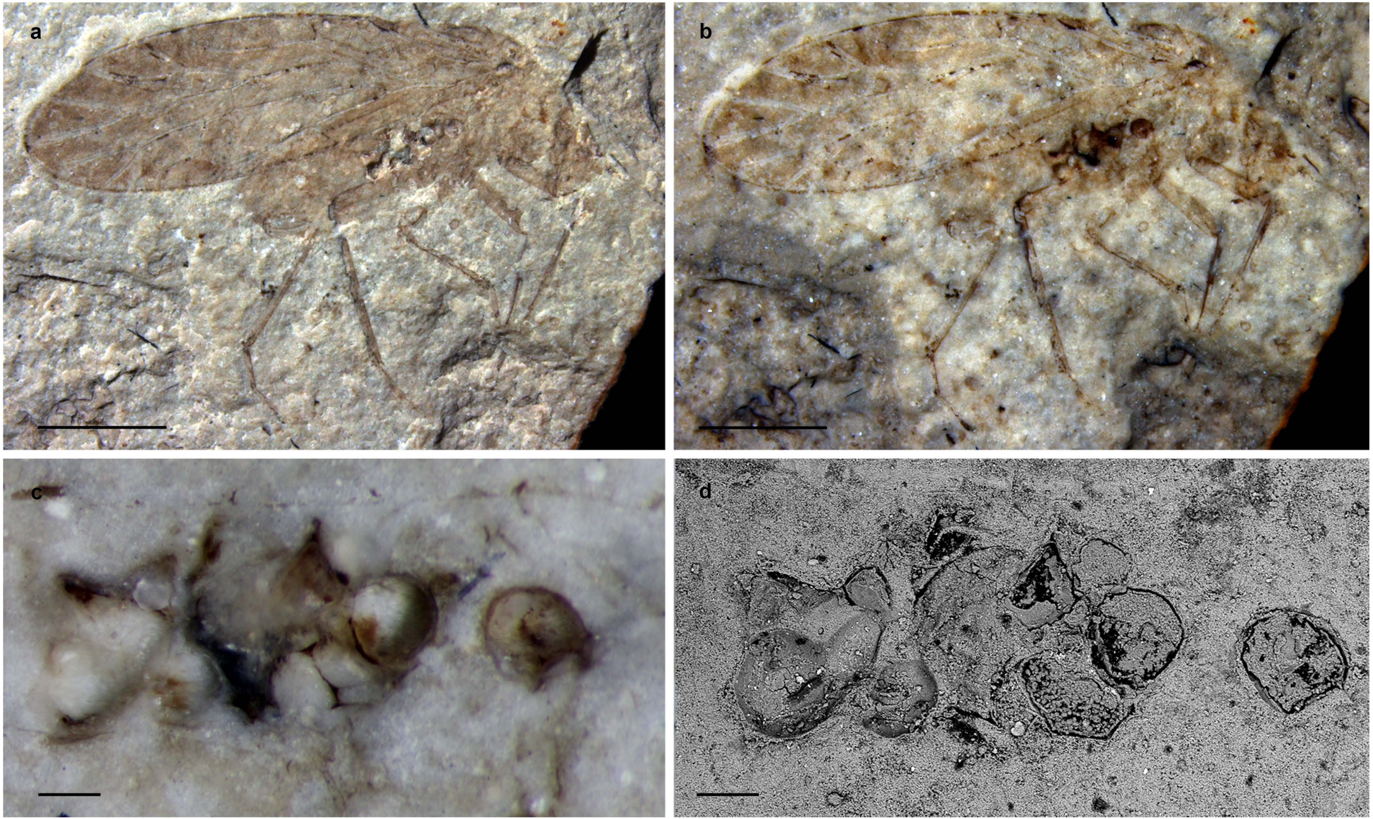
a
b
c
d

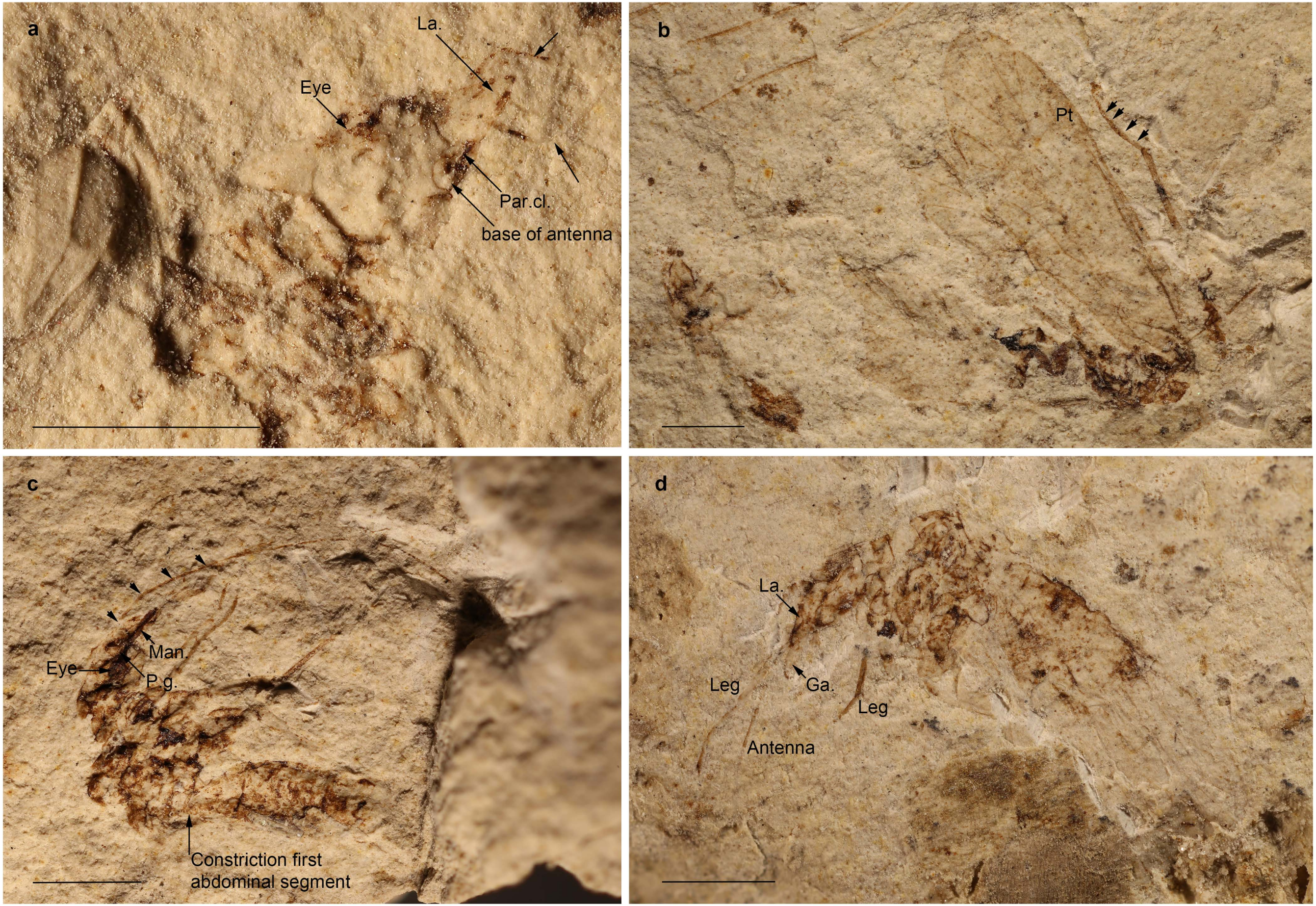
a
La.
Eye
Par.cl.
base of antenna
b
Pt
c
Man.
Eye
P.g.
Constriction first
abdominal segment
d
La.
Leg
Ga.
Leg
Antenna

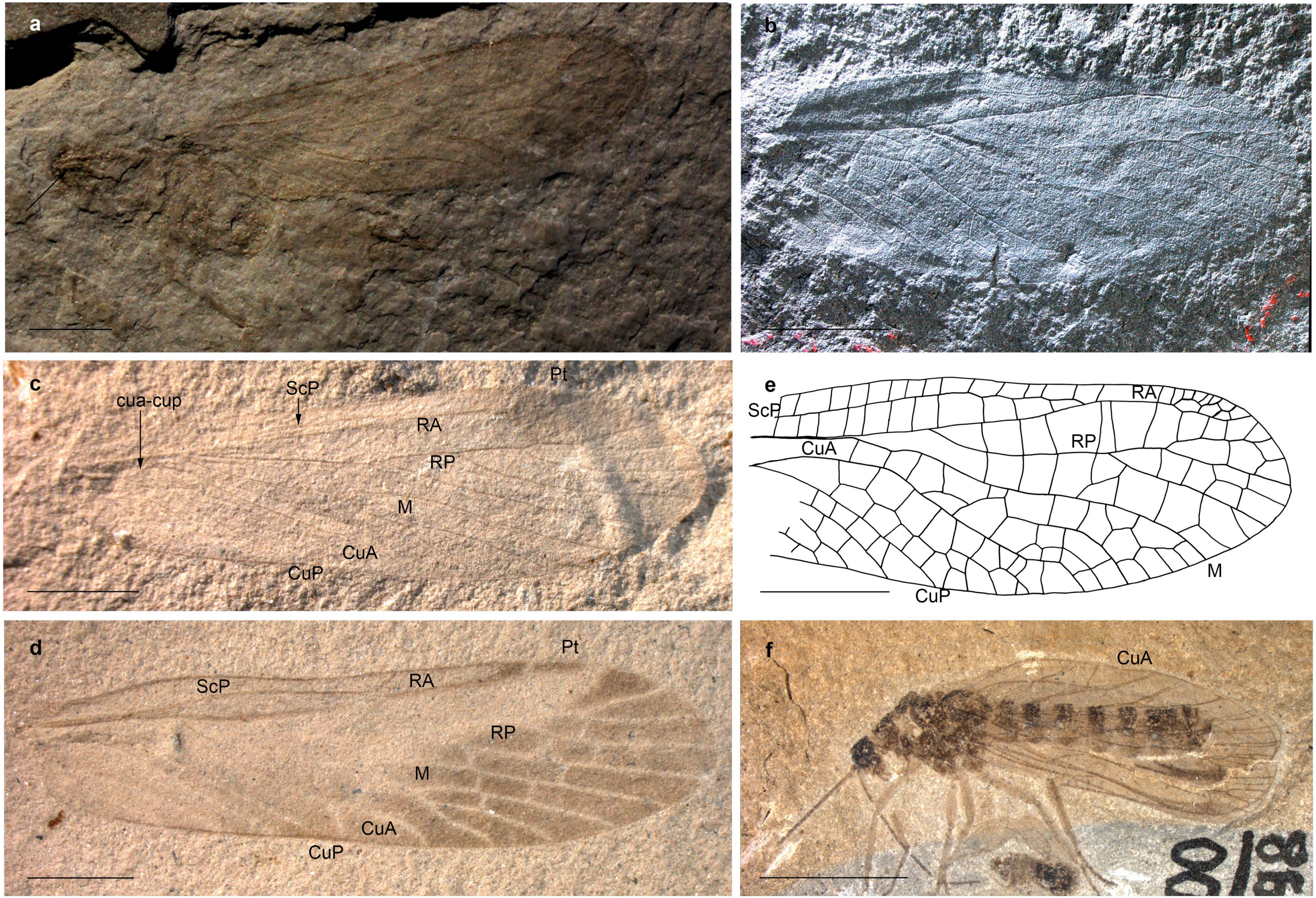

a
b
c
cua-cup
ScP
Pt
RA
RP
M
CuA
CuP
e
ScP
RA
CuA
RP
M
CuP
d
ScP
Pt
RA
RP
M
CuA
CuP
f
CuA

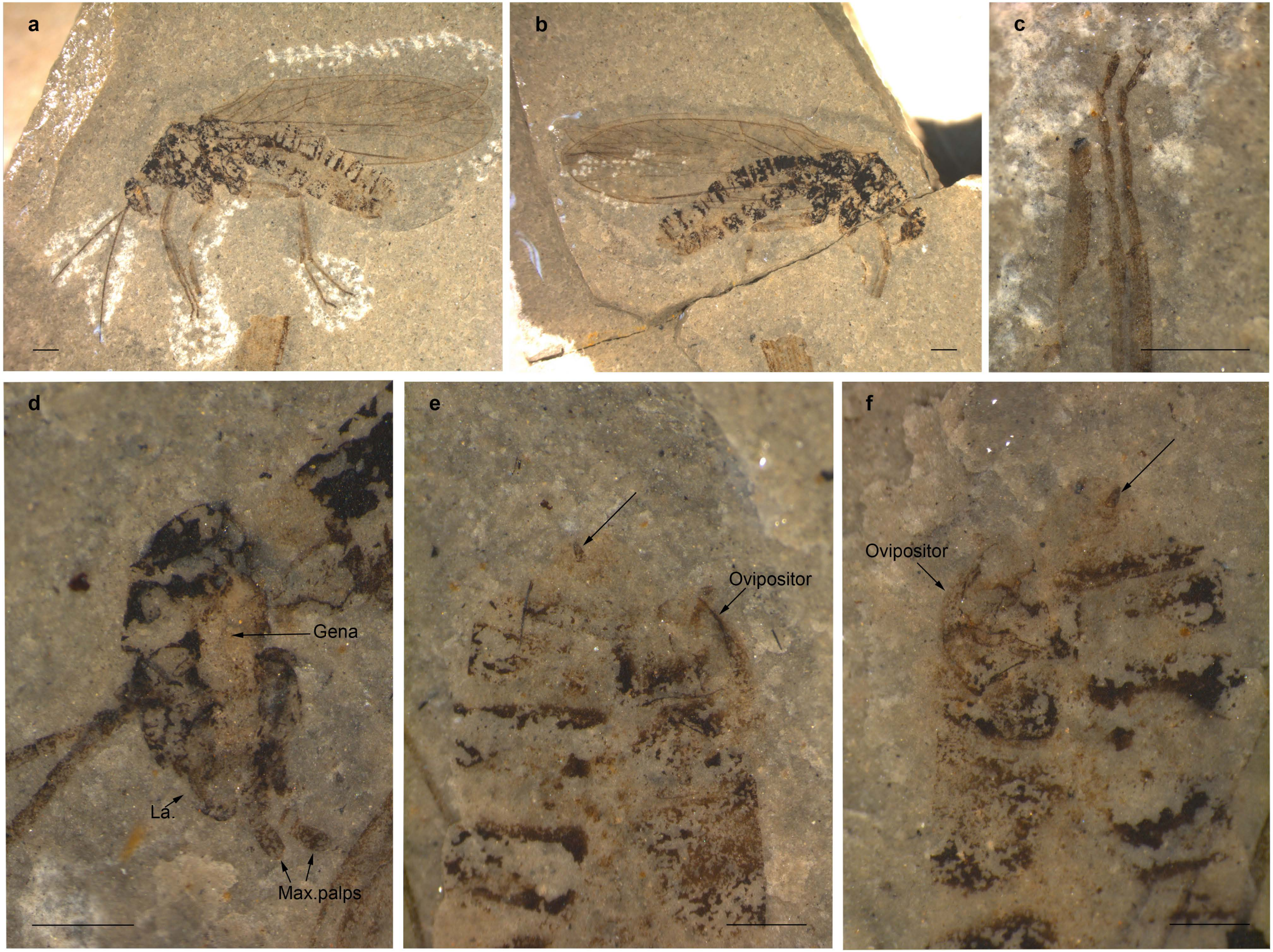
a
b
c
d
Gena
La.
Max.palps
e
Ovipositor
f
Ovipositor

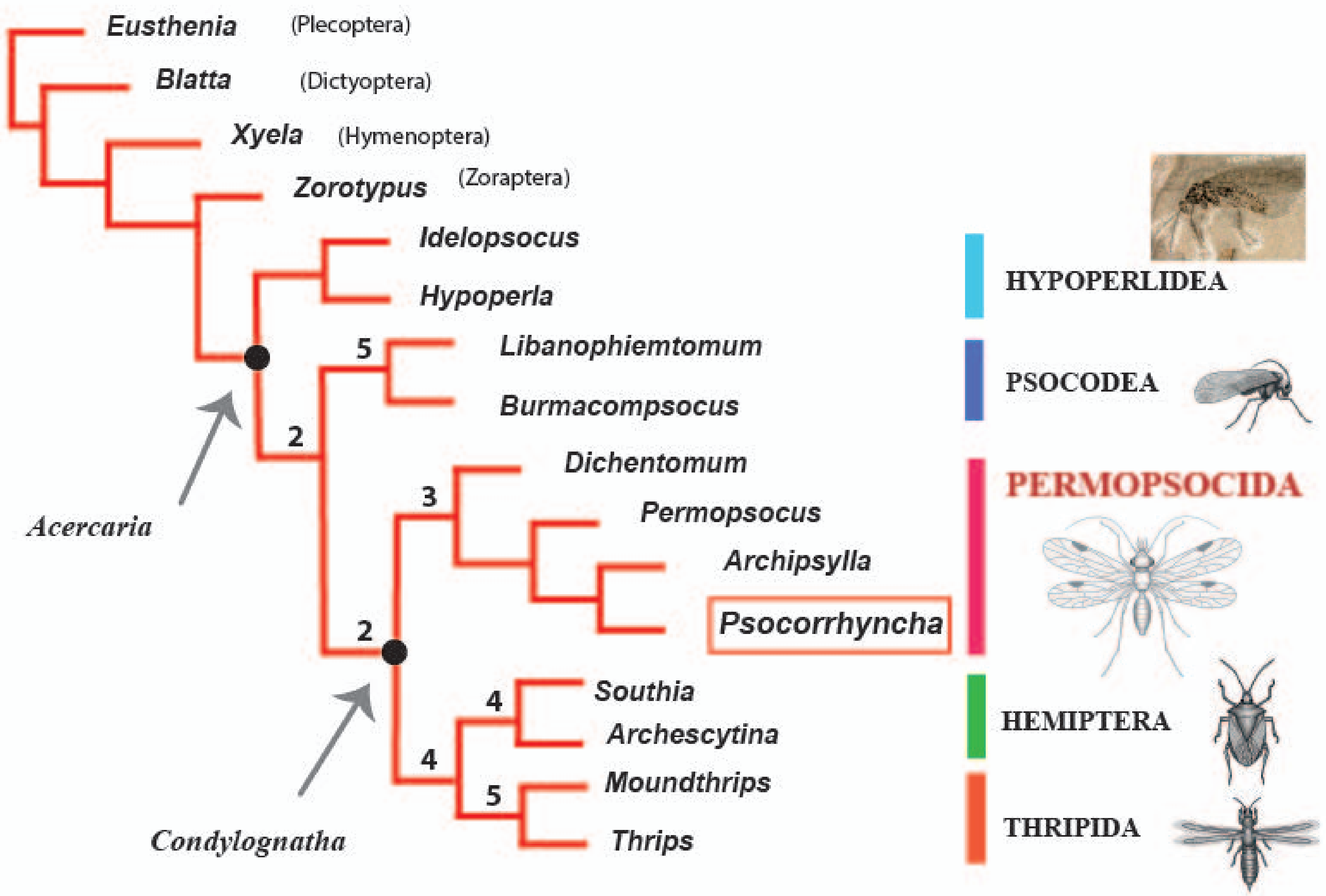

Eusthenia
(Plecoptera)
Blatta
(Dictyoptera)
Xyela
(Hymenoptera)
Zorotypus
(Zoraptera)
Idelopsocus
Hypoperla
5
Libanophiemtomum
Burmacompsocus
2
Acercaria
Dichentomum
3
Permopsocus
Archipsylla
Psocorrhyncha
2
Southia
4
Archescytina
4
Moundthrips
5
Thrips
Condylognatha
HYPOPERLIDEA
PSOCODEA
PERMOPSOCIDA
HEMIPTERA
THRIPIDA

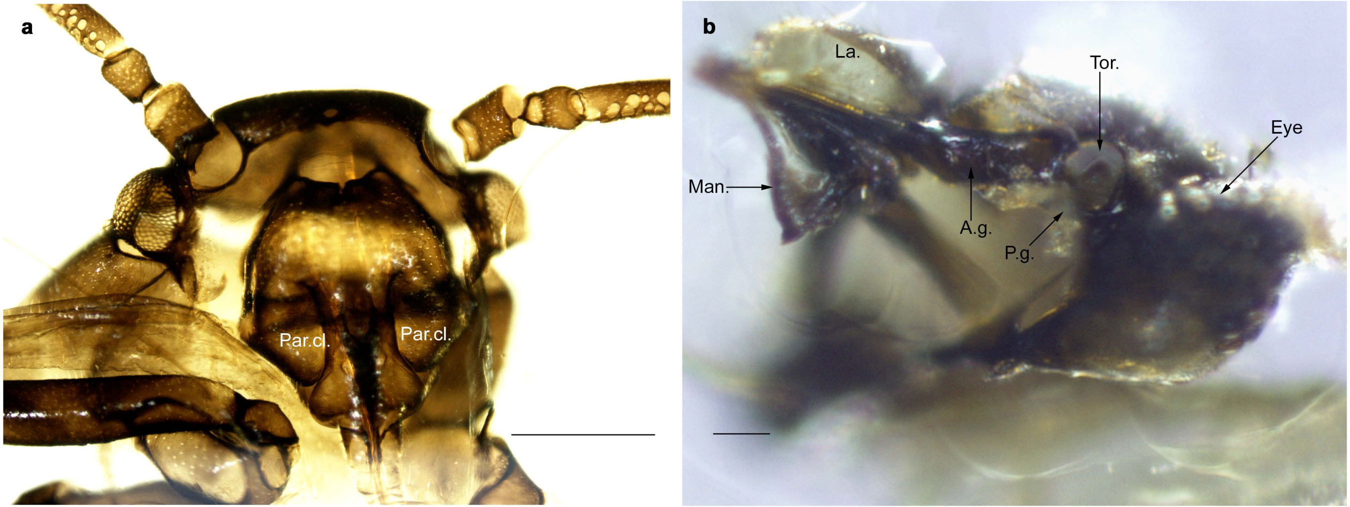
a
Par.cl.
Par.cl.
b
La.
Tor.
Eye
Man.
A.g.
P.g.
